# Methyl carbamates of phosphatidylethanolamines and phosphatidylserines reveal bacterial contamination in mitochondrial lipid extracts of mouse embryonic fibroblasts

**DOI:** 10.1038/s41598-023-40357-5

**Published:** 2023-08-26

**Authors:** Andrea Castellaneta, Vito Porcelli, Ilario Losito, Serena Barile, Alessandra Maresca, Valentina Del Dotto, Ludovica Sofia Guadalupi, Cosima Damiana Calvano, Valerio Carelli, Luigi Palmieri, Tommaso R. I. Cataldi

**Affiliations:** 1 Dipartimento di Chimica, Università degli Studi di Bari Aldo Moro, via Orabona 4, 70126 Bari, Italy; 2 Dipartimento di Bioscienze, Biotecnologie e Ambiente, Università degli Studi di Bari Aldo Moro, via Orabona 4, 70126 Bari, Italy; 3 Centro Interdipartimentale SMART, Università degli Studi di Bari Aldo Moro, via Orabona 4, 70126 Bari, Italy; 4 IRCCS Istituto delle Scienze Neurologiche di Bologna, Programma di Neurogenetica, via Altura 3, 40139 Bologna, Italy; 5 Dipartimento di Scienze Biomediche e Neuromotorie, Università degli Studi di Bologna, via Altura 3, 40139 Bologna, Italy; 6 CNR-Istituto di Biomembrane, Bioenergetica E Biotecnologie Molecolari, via Giovanni Amendola, 122/O, 70126 Bari, Italy

**Keywords:** Biochemistry, Chemistry

## Abstract

The occurrence of methyl carbamates of phosphatidylethanolamines and phosphatidylserines in the lipid extract of mitochondria obtained from mouse embryonic fibroblasts was ascertained by hydrophilic interaction liquid chromatography with electrospray ionization single and multi-stage mass spectrometry, performed using sinergically a high resolution (quadrupole-Orbitrap) and a low resolution (linear ion trap) spectrometer. Two possible routes to the synthesis of methyl carbamates of phospholipids were postulated and evaluated: (i) a chemical transformation involving phosgene, occurring as a photooxidation by-product in the chloroform used for lipid extraction, and methanol, also used for the latter; (ii) an enzymatic methoxycarbonylation reaction due to an accidental bacterial contamination, that was unveiled subsequently on the murine mitochondrial sample. A specific lipid extraction performed on a couple of standard phosphatidyl-ethanolamines/-serines, based on purposely photo-oxidized chloroform and deuterated methanol, indicated route (i) as negligible in the specific case, thus highlighting the enzymatic route related to bacterial contamination as the most likely source of methyl carbamates. The unambiguous recognition of the latter might represent the starting point toward a better understanding of their generation in biological systems and a minimization of their occurrence when an artefactual formation is ascertained.

## Introduction

The rapid expansion of MS-based lipidomics has led to cope with the inherent challenges related to the sample preparation and analysis, which might impair the reliable identification and/or quantification of lipids in complex samples. As recently reviewed by Hu et al.^1^, despite electrospray ionization (ESI), one of the two major ionization approaches used in lipidomics, along with matrix-assisted laser desorption ionization, is generally considered a *soft* ionization process, artefacts may result when specific lipids are ionized in the ESI source. If phospholipids (PL) are considered, phosphatidic acids (PA) can be generated from the *in-source* fragmentation of phosphatidylserines (PS), due to the neutral loss of the serine moiety in the polar head, whereas dimethyl-phosphatidylethanolamines (dimethyl-PE) can be originated through *in-source* fragmentation of negatively charged adducts formed by phosphatidylcholines (PC) with anions like acetate or formate, whose ammonium salts are often added to the mobile phase during chromatographic separations of PL^1^. Such artefacts can be revealed by hydrophilic interaction liquid chromatography followed by electrospray ionization mass spectrometry (HILIC-ESI–MS), thanks to its ability to separate PL based on their class^2, 3^. Indeed, the PA/PS and PE/PC couples can be separated in the time domain through an appropriate HILIC elution gradient (see, for example, Ref.^4^).

In the last ten years, HILIC-ESI–MS has been successfully employed in our laboratory to unveil further artefacts involving phospholipids, like those related to undesired enzymatic side reactions occurring during sample preparation. In particular, the artificial generation of *lyso*-phospholipids, resulting from the action of endogenous phospholipase A1/A2, was evidenced during lipid extraction from mussels, if proper deactivation steps for those enzymes were not introduced^5^. In a further investigation, the generation of PA and of their methyl esters from PC and phosphatidylglycerols (PG), through hydrolysis and trans-phosphatidylation reactions catalysed by endogenous phospholipase D, respectively, was evidenced during lipid extraction from microgreens using HILIC-ESI–MS^6^.

Recently, two unexpected chromatographic bands were evidenced in our laboratory during the HILIC-ESI-Fourier transform-MS (HIILC-ESI-FTMS) analysis of mitochondrial lipid extracts obtained from mouse embryonic fibroblasts (MEF). A careful evaluation of the underlying mass spectra, integrated by the acquisition and interpretation of targeted multi-stage (MS^n^, with n = 2–3) spectra on major detected ions, suggested that the two bands corresponded to the methyl carbamates of PE and PS (mc-PE/mc-PS), i.e., to PE and PS bearing a methoxycarbonylic moiety linked to the amino group of the polar head. Notably, such species were observed by Garrett et al*.*^7^ and Vyssotski et al*.*^8^ in bacterial and algal extracts, respectively, and interpreted as artefacts of the lipid extraction protocols, that involved the use of chloroform and methanol. Indeed, methyl carbamates can be generated as the products of an undesired side reaction between PL containing amino groups, phosgene occurring in photo-oxidated chloroform, and methanol, a process that was shown to occur also on drugs and metabolites including amine substituents^9^. However, the hypothesis of a phosgene-mediated side reaction seemed unable to explain the generation of ethyl carbamates of PE detected by Garrett et al*.* in *Escherichia coli* lipid extracts, because no ethanol was ever used in the extraction process^7^. To address this controversial issue, mc-PE and mc-PS detected in mitochondrial lipid extracts of MEF were carefully characterized in our laboratory by mass spectrometry, as it will be described in detail in the present paper. Some experiments were then performed to verify whether the phosgene-mediated chemical route to mc-PS and mc-PE generation might be the most likely one also in the present case. As also discussed in this paper, the occurrence of a bacterial contamination of murine mitochondrial samples was ascertained and found to be a better explanation for mc-PS and mc-PE formation. The information gained during the present study might thus be useful to understand how these unusual PL can be formed and which role they might play in biological systems when naturally generated.

## Results

### Mitochondrial samples characterization previous to lipidomic analysis

**Figure 1:**
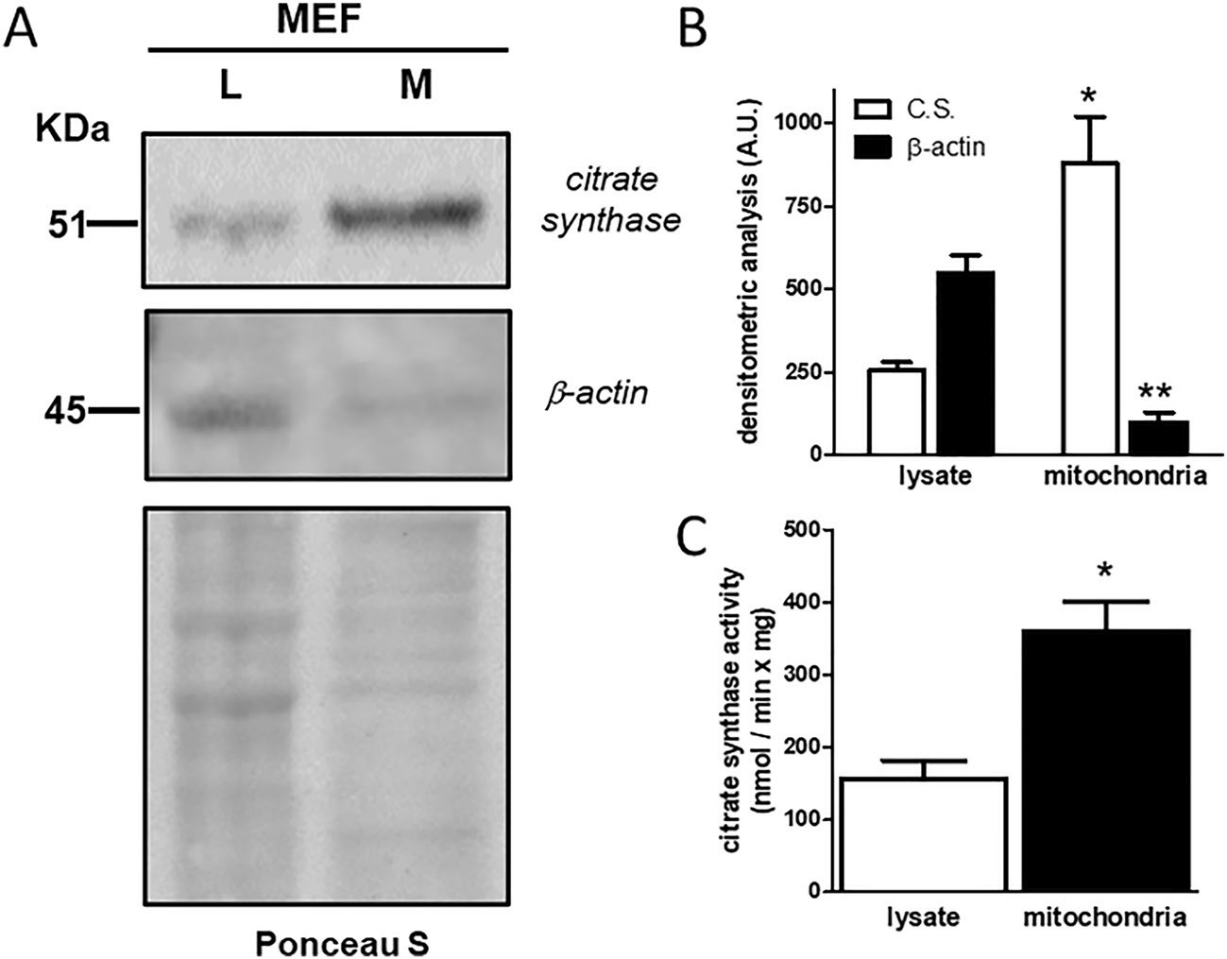
(**A**) Representative western blot of three indipendent experiments in which antibodies against a mitochondrial marker, citrate synthase, and a cytosolic protein, β-actin, were used on the cellular lysate (L) and the mitochondrial fraction (M) derived from a MEF cells line. As loading control, the membrane coloured with red Ponceau S before the immunodetection is also shown. (**B**) Citrate synthase (C.S.) and β-actin contents quantified by densitometric analysis of three indipendent experiments of western blot. Significant differences referred to cell lysate and mitochondria are marked with * and ** (p < 0.01), respectively. (**C**) Citrate synthase activity, expressed as initial rate, measured in cell lysate and relative mitochondria; average and S.D. referred to three experiments are reported (* indicates a statistically significant difference, with p < 0.01). See Fig. S1 in the Supplementary Information for further details.

The purity and the enrichment of mitochondria from whole MEF was preliminarily assessed by western blot analysis measuring cytosolic (β-actin) and mitochondrial (citrate synthase) marker proteins. A representative western blot of total extract and enriched mitochondrial fraction of MEF is reported in Fig. 1A (see Fig. S1 in the Supplementary Information for further details).The densitometric analysis of the bands detected in three different experiments shows that the mitochondrial fraction (M) was enriched fourfold, in terms of citrate synthase, compared to cellular lysate (L), while cytosolic contamination, in terms of β-actin, was very low (Fig. 1B). This result confirmed that the mitochondria used for lipidomic analysis were pure, but also supported the idea that a pool of β-actin is localized in mitochondria, as demonstrated by Xie et al*.*^10^. However, how β-actin is organized inside mitochondria and whether it plays a functional role is not clear. Moreover, the activity of citrate synthase, an enzyme of the TCA cycle, was measured. As shown in panel C of Fig. 1, the specific activity for this enzyme was significantly increased during the purification of mitochondria from cells, thus confirming the results obtained by western blot analysis.

### Detection of two uncommon phospholipid classes in the mitochondrial lipid extracts of mouse embryonic fibroblasts

**Figure 2:**
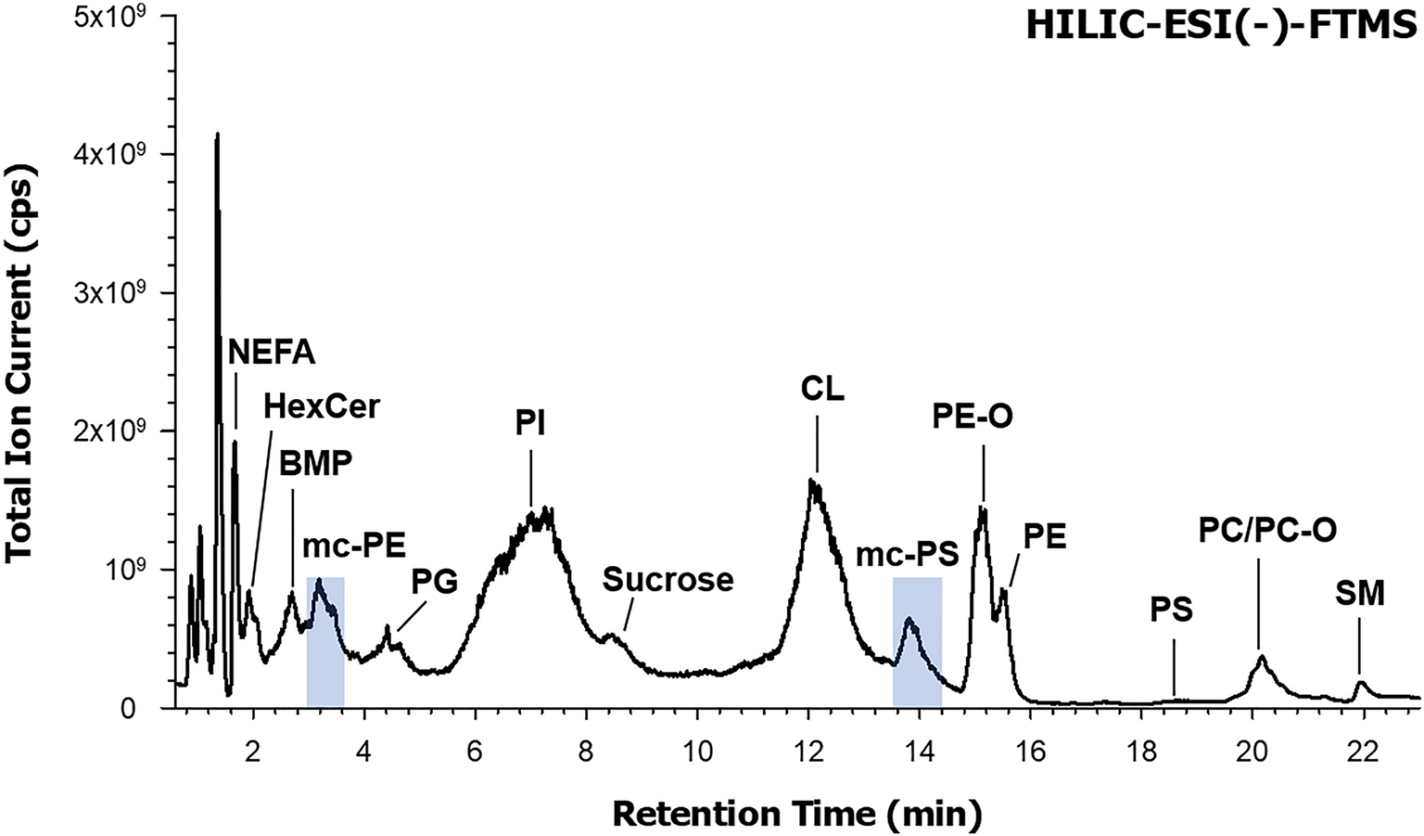
Total Ion Current (TIC) chromatogram resulting from the HILIC-ESI(−)-FTMS analysis of the lipid extract of a sample of mitochondria obtained from mouse embryonic fibroblasts (MEF). Shaded chromatographic bands correspond to lipids that were finally identified as the methyl carbamates of phosphatidylethanolamines (mc-PE) and phosphatidylserines (mc-PS). Legend for further lipids identified: non-esterified fatty acids (NEFA), hexosyl-ceramides (HexCer), bis-monoacylglycerophosphates (BMP), phosphatidylglycerols (PG), phosphatidylinositols (PI), cardiolipin (CL), phosphatidylcholines (PC) and sphingomyelins (SM).

The first clue for the presence of uncommon PL species in the lipid extract of a mitochondrial sample of MEF cells was obtained from the corresponding HILIC-ESI(−)-FTMS Total Ion Current (TIC) chromatogram, reported in Fig. 2; as it can be seen, several chromatographic bands were observed, each related to a specific lipid class. The band broadening often observed was related to the occurrence of a wide range of PL species of the same class, eluting at similar retention times. The FTMS spectrum averaged under each chromatographic band was carefully explored to retrieve the accurate *m/z* values of relevant peaks, which were used to search for lipid species using the *Online Lipid Calculator* (http://www.mslipidomics.info/lipid-calc/), after setting a ± 0.005 tolerance for *m/z* matching. As a result, ten lipid classes were recognized, corresponding to (in order of retention time): non-esterified fatty acids (NEFA), hexosyl-ceramides (HexCer), bis-monoacylglycerophosphates (BMP), phosphatidylglycerols (PG), phosphatidylinositols (PI), cardiolipins (CL), phosphatidylethanolamines (PE), phosphatidylserines (PS), phosphatidylcholines (PC), and sphingomyelins (SM). Despite the expected abundance in mitochondria^11^, PC exhibited a relatively weak band because negative ionization is less favourable for this class of PL, compared to PG, PI and CL, which are easily deprotonated. Notably, the PE band was recognized as the overlap of two partially resolved peaks, one of which was subsequently assigned to diacylic species (labelled as PE) and the other to alkylic-acylic species (generally labelled as PE-O), whose presence is well established in the mitochondrial samples^11^. The same combination of diacylic and alkylic-acylic species was recognized for PC, although they eluted under the same chromatographic band (see Fig. 2). Additionally, a weak band eluting between 8 and 9 min was related to sucrose, detected as a deprotonated molecule ([C_12_H_22_O_11_-H]^−^); its occurrence was likely related to its use in the mitochondrial isolation procedure and its subsequent partial co-extraction with lipids.

Notably, no correlation with lipid ions generated by the *Online Lipid Calculator* could be found for *m/z* values retrieved from FTMS spectra averaged under chromatographic peaks eluting in the retention time intervals 3.2–3.6 min and 13.5–14.4 min (see shaded peaks in Fig. 2). Nonetheless, tandem MS spectra collected for the major peak signals detected in those spectra exhibited couples of signals corresponding to carboxylate anions of fatty acyl chains (vide infra). It was then conjectured that the *m/z* values were related to PL having a modified polar head, thus eluting at retention times different from those of conventional PL. A systematic comparison between FTMS spectra averaged under the two unexpected chromatographic bands and those referred to bands of conventional PL, followed by targeted MS/MS and MS^3^ analyses, was thus undertaken to obtain further information.

### Identification of methyl carbamates of phosphatidylserines

**Figure 3:**
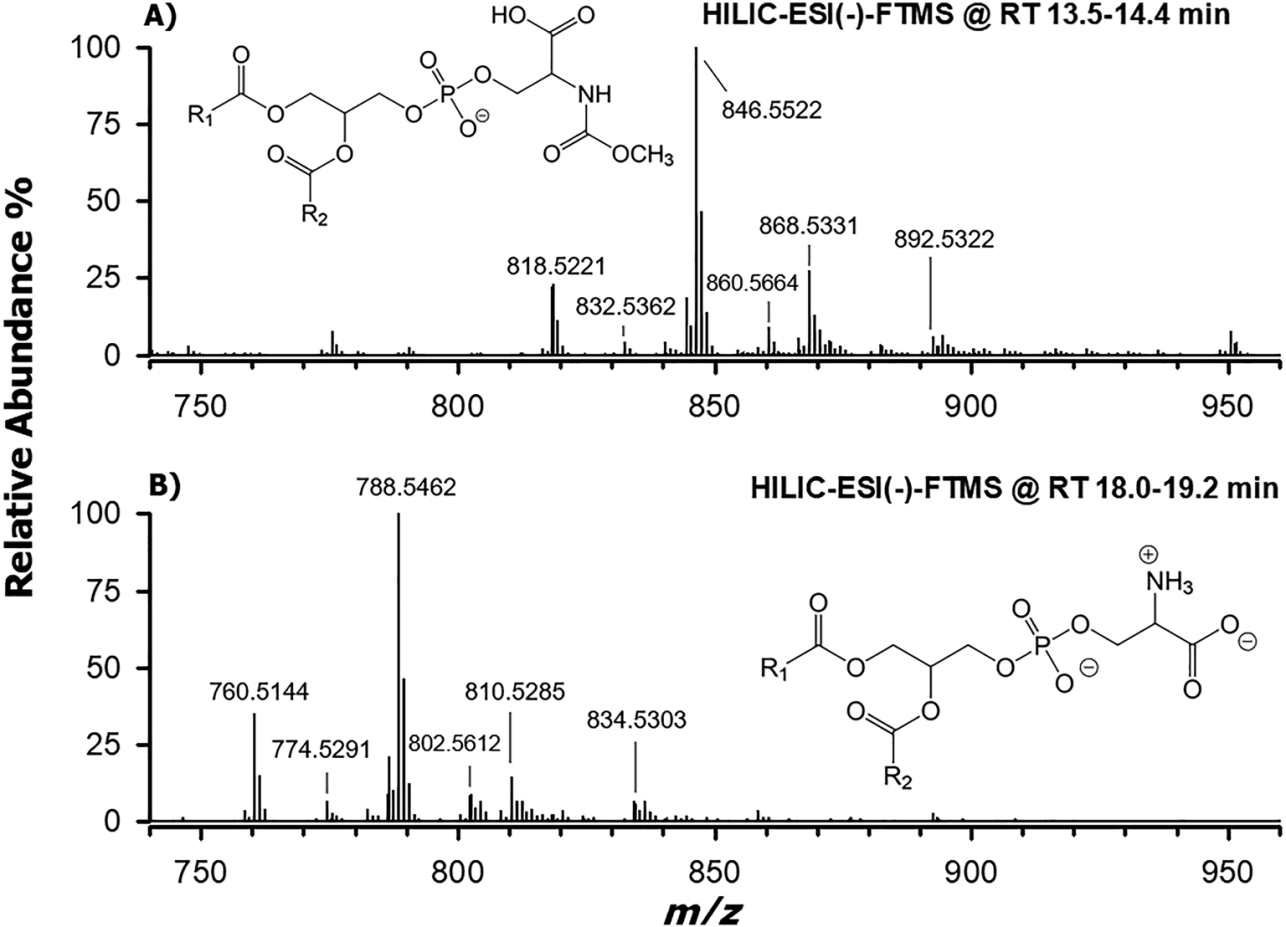
ESI(−)-FTMS spectra averaged under the bands assigned to mc-PS (**A**) and PS (**B**) detected in the TIC chromatogram shown in Fig. 1. The labels refer to accurate *m/z* ratios measured for the most intense peak signals. The general structures for the [M−H]^−^ ions of mc-PS and PS (the latter being reported as the deprotonated form of zwitterionic PS) are also shown.

As illustrated in Fig. 3, apart from being shifted towards higher *m/z* values, the ESI(−)FTMS spectrum averaged under the peak eluting between 13.5 and 14.4 min (panel A) exhibited a striking similarity with the one referred to the PS band (18.0–19.2 min, see panel B). The *m/z* shift observed between corresponding peak signals in the spectra (like, for example, the respective base peaks, detected at *m/z* 846.5522 and 788.5462) was calculated and its average value was 58.0052 ± 0.0019 units, compatible with the addition of a C_2_H_2_O_2_ formula (exact monoisotopic mass: 58.0055) to the chemical formula of PS. Notably, the base peak at *m/z* 788.5462 of plot B was assigned to the [M−H] ion of PS 36:1 (exact *m/z* 788.5447), with M representing the zwitterionic form (see the molecular structure in Fig. 3B). A signal referred to the [M-2H+Na]^−^ adduct of PS 36:1 (experimental *m/z* 810.5285), already described in the literature^12^ and likely generated by the presence of sodium ion traces in the HILIC mobile phase, was also detected (see Fig. 3B). Interestingly, the same ionization pattern was observed for the PS 36:1 putatively modified by the additional C_2_H_2_O_2_ moiety, with peak signals detected at *m/z* 846.5522 and 868.5331, corresponding to [M−H]^−^ and [M-2H+Na]^−^ ions, respectively (see Fig. 3A).

#### MS/MS and MS^3^ analysis of [M−H]^−^ ions related to methyl carbamates of phosphatidylserines

**Figure 4:**
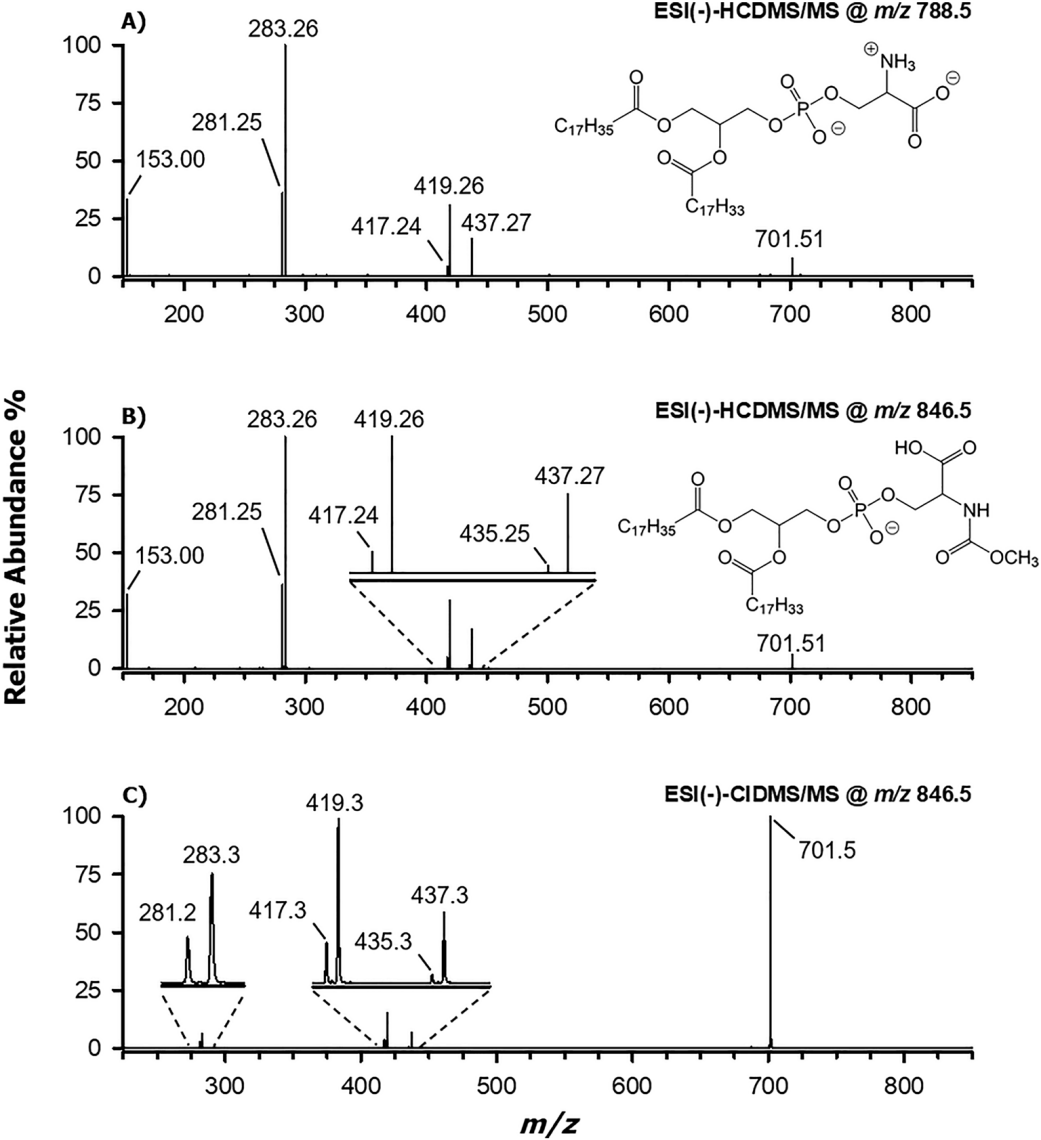
ESI(−)-HCD-MS/MS spectra (Normalized Collisional Energy, NCE = 28) obtained for the [M−H]^−^ ions of: (**A**) PS 18:0/18:1 (*m/z* 788.55) and (**B**) mc-PS 18:0/18:1 (*m/z* 846.55), whose structures are also reported. (**C**) ESI(−)-CID-MS/MS spectrum (NCE = 35) obtained for the [M−H]^−^ ion of mc-PS 18:0/18:1. See the text for the assignments of the detected peak signals.

To address the findings resulting from MS acquisitions, high-resolution MS/MS spectra and low-resolution MS/MS and MS^3^ spectra were acquired on the not-yet classified PS derivatives. The former ones were obtained using a quadrupole-Orbitrap mass spectrometer equipped with a High energy Collisional Dissociation (HCD) cell for ion fragmentation; the latter were acquired through Collisional Induced Dissociation (CID), performed using a linear ion trap mass spectrometer (see the “Methods” section for details). As an example, the HCD-MS/MS spectra of [M−H]^−^ ions related to PS 36:1 (*m/*z 788.55) and to its modified counterpart (*m/*z 846.55) are compared in Fig. 4, panels A and B, respectively. Note that, due to the lower resolving power adopted for the FTMS/MS analyses, compared to FTMS acquisitions, experimental *m/z* values and those referred to precursor ions were rounded off to the second decimal digit. In both cases, a product ion at *m/*z 701.51, corresponding to a deprotonated PA with a sum composition of 36:1, was obtained. Since a PA is formed upon the loss of dehydrogenated serine from a PS, this was direct evidence of the fact that the additional C_2_H_2_O_2_ moiety was embedded into the serine moiety of the modified PS, thus it was lost upon fragmentation, leading to the [M−H]^−^ ion of PA 36:1. Not surprisingly, both tandem MS spectra shared all the other detected product ions, namely those with *m/z*: (i) 281.25 and 283.26, corresponding to the anions of fatty acids 18:1 and 18:0, respectively; (ii) 419.26 and 417.24, arising from the neutral loss as fatty acids of the two acyl chains; (iii) 437.27 and 435.25 (the latter signal, very weak, can be seen only in the expanded plot in Fig. 4B), arising from the neutral losses of acyl chains 18:1 and 18:0 as ketenes, respectively (see Fig. S2 in the Supplementary Information). As reported by Hsu and Turk^13^, the prevalence of the neutral losses of acyl chains as fatty acids over those of ketenes is a typical feature of PA fragmentation. Moreover, based on the relative abundance of peak signals related to acyl chain neutral losses and on another rule of PA fragmentation, i.e., the prevalence of fatty acid and ketene loss for the chain linked to the sn-2 position of glycerol^13^, the regiochemistry of the PS 36:1 and of its modified derivative, i.e., the location of the acyl chains on the sn_1_/sn_2_ positions of the glycerol backbone, was assessed as 18:0/18:1. The HCD-MS/MS spectrum was completed by a signal at *m/z* 153.00, corresponding to a glycerocyclophosphate anion and arising from the loss of both acyl chains, one as fatty acid and the other as ketene (see Fig. S2). As shown in Fig. 4C, the CID-MS/MS spectrum of the precursor ion at *m/z* 846.5, i.e., the PS 36:1 derivative, was consistent with the HCD-MS/MS spectrum, excepting for the relative abundances of product ions. As expected, due to the lower collisional energy of CID, a much less relevant fragmentation of the anion of PA 36:1 (*m/z* 701.5) occurred. The *m/z* 153.00 ion was likely not detected in this case due to the low mass cut-off imposed by the linear ion trap mass spectrometer.

#### MS/MS and MS^3^ analysis of [M-2H+Na]^−^ ions related to methyl carbamates of phosphatidylserines

**Figure 5:**
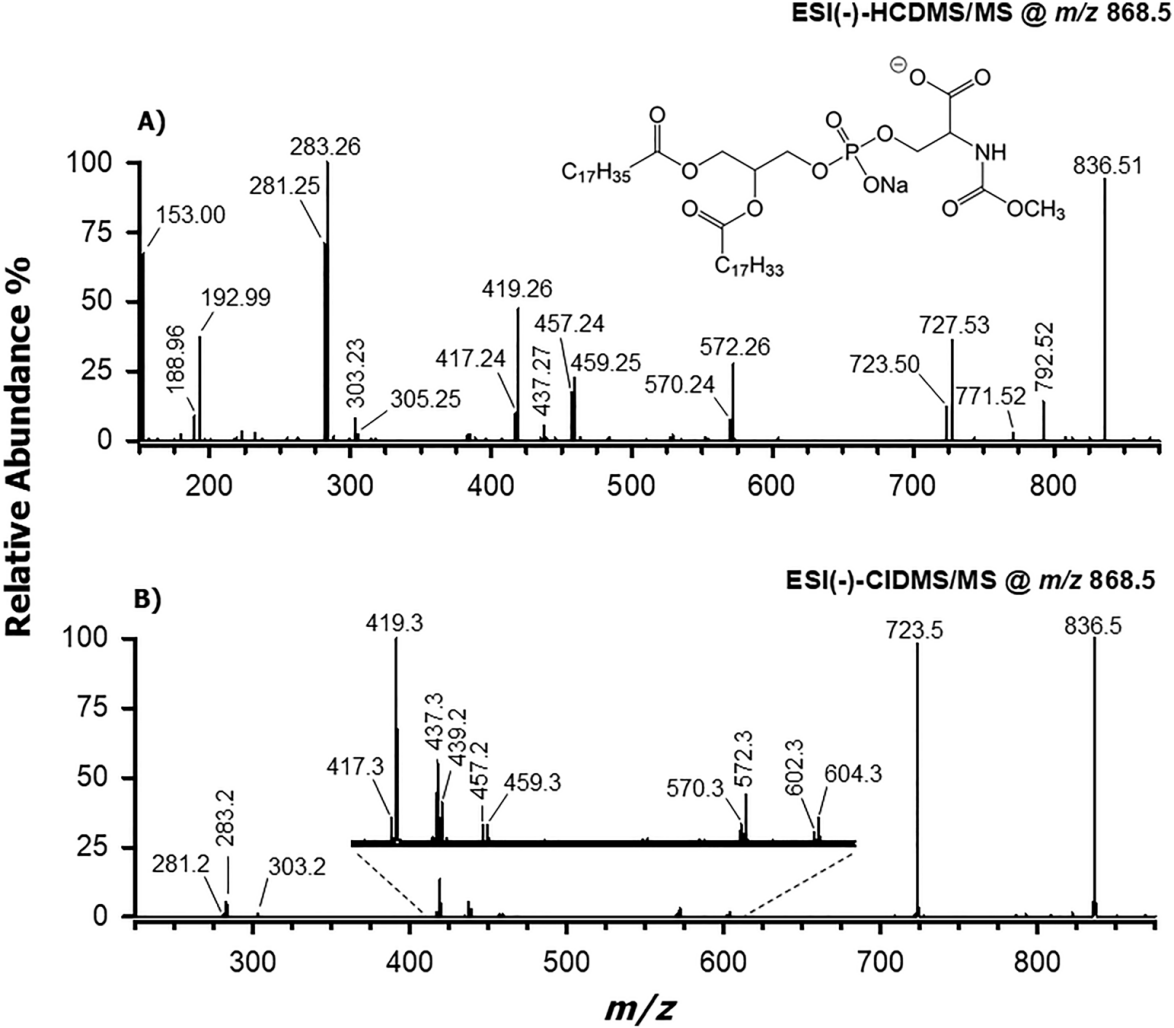
(**A**) ESI(−)-HCD-MS/MS spectrum (NCE = 28) and (**B**) CID-ESI(−)-MS/MS spectrum (NCE = 35) obtained for the [M-2H+Na]^−^ ion of PS 18:0/18:1. The precursor ion structure is also reported.

Although tandem MS spectra of deprotonated molecules ([M−H]^−^) were not useful to explain how the serine moiety was modified in the PS derivatives, this information could be retrieved by fragmenting the corresponding [M-2H+Na]^−^ ions. HCD-MS/MS and CID-MS/MS spectra obtained for the modified PS 36:1, at *m/z* 868.53/868.5, are thus reported, respectively, in panels A and B of Fig. 5. The modification that occurred on the PS species was inferred from the diagnostic product ion at *m/z* 836.53/836.5 detected in HCD/CID MS/MS spectra. As illustrated in Fig. S3 of Supplementary Information, these values were compatible with the neutral loss of methanol (32.0262 Da), in turn suggesting that a methoxy-carbonylic moiety was linked to the serine nitrogen atom of the modified PS 36:1 (see the structure reported in Fig. 5). Notably, this modification is consistent with the introduction of a C_2_H_2_O_2_ moiety in the minimal formula of PS species. The loss of methanol, involving a 1,3 H transfer from the serine *N* atom to the methoxy group *O* atom, led to a stable isocyanate functionality in the resulting product ion (see Fig. S3). Subsequent ketene losses of both acyl chains were observed, leading to product ions with *m/z* values 570.24 and 572.26; for the sake of clarity, only the latter process is depicted in Fig. S3. These product ions were detected under both collisional energy regimes (their *m/z* values being 570.3 and 572.3 in the CID-MS/MS spectrum), although they were much more abundant in the HCD-MS/MS spectrum (see Fig. 5A). Different pathways could be hypothesised for further fragmentations of the ion detected at *m/z* 836.53, according to the location of the Na^+^ ion in its chemical structure. Indeed, when Na^+^ was coupled with the deprotonated carboxylic group, product ions at *m/z* 792.51, 771.52 and 727.53 were generated, at least under HCD conditions (see Fig. 5A). The occurrence of a weak peak signal at *m/z* 771.52 ion most likely suggests the loss of sodium isocyanate (NaOCN, 64.9878 Da), thus indirectly confirming the presence of a methoxy-carbonylic moiety linked to the serine NH_2_ group in the precursor ion. Notably, the three product ions were detected also in the CID-MS^3^ spectrum acquired on the *m/z* 836.5 ion resulting from fragmentation of the *m/z* 868.5 precursor ion (see Fig. S4A in the Supplementary Information). In the same spectrum, a product ion at *m/z* 529.3 was also detected. This ion can be explained through the subsequent neutral losses of the 18:1 acyl chain as ketene (see the peak signal at *m/z* 572.3 in Figs. S3 and S4A) and isocyanic acid (HOCN, 43.0058 Da). Following an analogous pathway (not reported in Fig. S3), i.e., the loss of the 18:0 acyl chain as a ketene (*m/z* 570.3) along with isocyanic acid, the *m/z* 527.2 ion, also detected in the 868.5 > 836.5 > MS^3^ spectrum was generated (see Fig. S4A).

Several product ions detected in both HCD-MS/MS and CID-MS/MS spectra of the mc-PS 18:0/18:1 [M-2H+Na]^−^ ion arose from fragmentation pathways starting from the *m/z* 723.50/723.5 ion (i.e., the [M-2H+Na]^−^ ion of PA 18:0/18:1, with an exact *m/z* value 723.49). The latter was generated from the precursor ion through a neutral loss in the gas phase involving the modified serine moiety, as evidenced in Fig. S3. A similar process was hypothesised in the scheme to explain the generation of the product ion at *m/z* 723.5 also from the *m/z* 836.5 ion (see Fig. 5B), a process confirmed by the detection of the *m/z* 723.5 ion in the 868.5 > 836.5 > MS^3^ spectrum (see Fig. S4A).

As shown in Fig. S3, different types of neutral losses of acyl chains from the *m/z* 723.5 ion, i.e., as ketenes, sodium carboxylates or even aldehydes, sometimes occurring consecutively, were hypothesised to explain most product ions detected in HCD and CID tandem MS spectra of mc-PS 18:0/18:1. Many of them could be identified also in the 868.5 > 723.5 > MS^3^ spectrum, reported in Fig. S4B. Carboxylates of the 18:0 and 18:1 chains were also detected, thus providing a further confirmation of the acyl chains identity in the precursor ion.

A final consideration is deserved by peak signals detected at *m/z* 808.5 and 743.5 in the 868.5 > 836.5 > MS^3^ spectrum (see Fig. S4A). As emphasized in Fig. S5 (Supplementary Information), the former was likely generated upon carbon monoxide neutral loss from the polar head of the *m/z* 836.5 ion. In turn, the product ion at *m/z* 743.5 could be formally generated by the *m/z* 808.5 one, and the only possible interpretation of this process would be the neutral loss of sodium isocyanate, further confirming the methoxy-carbonylation of the NH_2_ group of PS 18:0/18:1.

The fragmentation behaviour of mc-PS 18:0/18:1 described so far was systematically observed for all major ions detected in the FTMS spectrum averaged under the 13.5–14.4 min chromatographic band, thus suggesting that all PS occurring in the analyzed mitochondrial lipid extract underwent a partial conversion into methyl carbamates. Two possible explanations for this process will be discussed later.

### Identification of methyl carbamates of phosphatidylethanolamines

A remarkable similarity in signal profiles was observed between the FTMS spectra averaged under the PE chromatographic band (retention time 14.8–16.0 min) and the one detected between 2.8 and 3.6 min (see Fig. S6 of the Supplementary Information), with a + 58 units shift found in the corresponding nominal *m/z* values. This difference, analogous to the one mentioned between PS and mc-PS, suggested that also the NH_2_ groups of PE and PE-O might be involved in the methoxy-carbonylation process.

**Figure 6:**
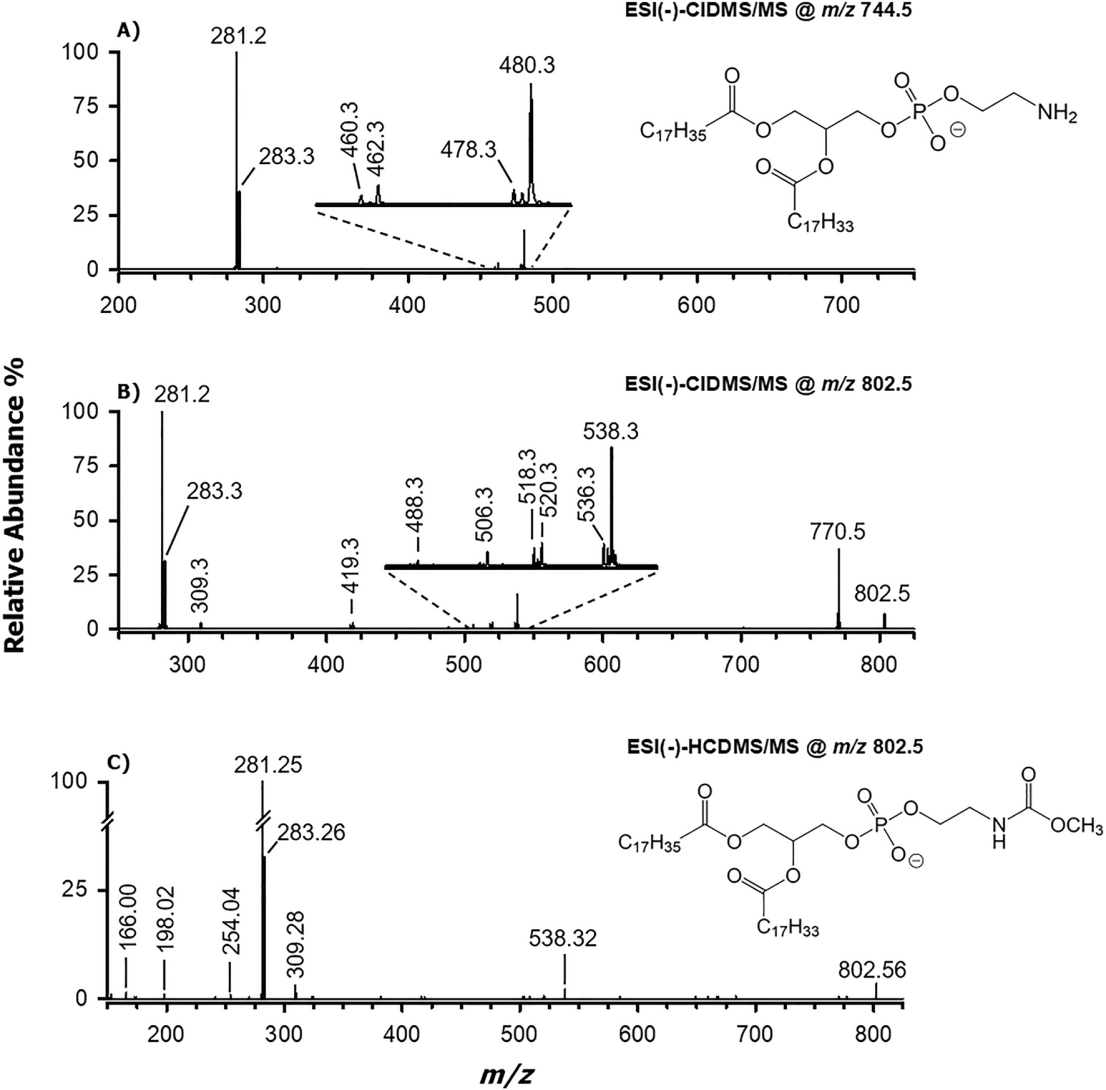
ESI(−)-CID-MS/MS spectra (NCE = 35) obtained for the [M−H]^−^ ions of: (**A**) PE 18:0/18:1 (*m/z* 744.5) and (**B**) mc-PE 18:0/18:1 (*m/z* 802.5), whose structures are reported as insets to panels (**A**) and (**C**). (**C**) ESI(−)-HCD-MS/MS spectrum (NCE = 28) obtained for the [M−H]^−^ ion of mc-PE 18:0/18:1. See the text for the assignments of the detected peak signals.

Evidence supporting this hypothesis was obtained by performing MS^n^ analyses on the [M−H]^−^ ions of the most abundant putative mc-PE species and of their PE precursors. As an example, CID-MS/MS spectra obtained for ions detected at *m/*z 744.5 and 802.5, corresponding to PE 36:1 and its methoxy-carbonylated counterpart, respectively, are shown in Fig. 6. The tandem MS spectrum of the *m/z* 744.5 ion was dominated by peak signals related to the carboxylates of fatty acids at *m/z* 281.2 and 283.3, clearly suggesting the presence of 18:1 and 18:0 side chains, respectively. Signals related to side-chain neutral losses indicated ketene losses (*m/z* 480.3 and 478.3, see the inset to Fig. 6A) as prevailing over fatty acid ones (*m/z* 462.3 and 460.3), as expected for a PE species^14^. Their relative abundance was useful to infer the regiochemistry as 18:0/18:1, in agreement with the well-known prevalence, first reported by Hsu and Turk^14^, of ketene loss from the sn-2 position of glycerol when negative ions of PE are fragmented. Peak signals referred to the anions of 18:1 and 18:0 fatty acids prevailed also in the CID-MS/MS spectrum of putative mc-PE 36:1 (*m/z* 802.5) and those related to ketene losses (*m/*z 538.3 and 536.3) respected the gas-phase fragmentation rules typical of PE, prevailing over signals related to fatty acid losses (*m/z* 520.3 and *m/z* 518.3).

The CID-MS/MS spectrum of the putative mc-PE 18:0/18:1 exhibited a relatively intense peak signal at *m/z* 770.5 (see Fig. 6B), compatible with the -32 units shift in nominal *m/z* related to the methanol neutral loss already discussed for mc-PS. The same process could be invoked also in the case of product ions resulting from acyl chains losses as ketenes or fatty acids (see peak signal couples at *m/z* 520.3/488.3 and 538.3/506.3 ion in the inset to Fig. 6B). As emphasized by the 802.5 > 770.5 > MS^3^ spectrum, reported in Fig. S7A (Supplementary Information), the release of fatty acid carboxylates and the ketene/fatty acid neutral losses occurred also from the [M−H]^−^ ion of mc-PE 18:0/18:1, after the methanol loss. Moreover, two low abundance ions were detected in the CID MS/MS spectrum of mc-PE 18:0/18:1, at *m/z* 419.3 and 309.3 (see Fig. 6B). As illustrated in the general fragmentation pattern reported in Fig. S8, the former was the result of three consecutive neutral losses: methanol (770.5), 18:1 chain ketene (506.3) and N-(2-hydroxyethyl)-isocyanate. The last process provided additional proof of the methoxy-carbonylation that occurred on the NH_2_ group of PE 18:0/18:1; notably, the correlation between the final fragment at *m/z* 419.3 and the one at *m/z* 770.5 was confirmed by the 802.5 > 770.5 > MS^3^ spectrum, shown in Fig. S7A. Moreover, an *m/z* 419.3 ion was detected also in the 802.5 > 538.3 MS^3^ spectrum (see Fig. S7B), i.e., during the gas-phase fragmentation of the product ion resulting from the 18:1 ketene loss. With the accurate value (309.28) obtained from the HCD-MS/MS spectrum acquired on the *m/z* 802.55 ion (see Fig. 6C), the product ion at *m/z* 309.3 was finally assigned as the carboxylate anion of a 20:1 fatty acid. Most likely, the latter arose from the fragmentation of mc-PE 16:0_20:1, which is the methyl carbamate of a minor PE, a co-eluted isomer of the mc-PE 18:0/18:1.

The HCD-MS/MS spectrum of mc-PE 18:0/18:1 (see Fig. 6C) showed additional, yet weak, peak signals in the low *m/z* range. Among them, the one at *m/z* 254.04 ion was interpreted as the result of the sequential losses of the 18:1 chain as ketene and the 18:0 chain as fatty acid, as suggested in Fig. S8. This interpretation was corroborated by the detection of the same ion (*m/z* 253.9) in the CID-MS^3^ spectrum referred to the mc-PE 18:0/18:1 fragment resulting from the 18:1 ketene loss (*m/z* 538.3, see Fig. S7B). Interestingly, the *m/z* 253.9 ion was also able to release methanol, leading to the product ion at *m/z* 221.9. The 18:0 chain loss as a ketene from the precursor ion at *m/z* 538.3 led to the *m/z* 272.1 fragment (see Fig. S7B). The same network of fragmentations was inferred for the product ions of mc-PE 18:0/18:1 upon loss of the 18:0 ketene (*m/z* 536.3 ion, see Fig. S7C). However, for the sake of clarity, only the fragmentation pathways of the *m/z* 538.3 ion were reported in Fig. S8.

As for product ions detected at *m/z* 198.02 and 166.00 in the HCD-MS/MS spectrum of mc-PE 18:0/18:1 (see Fig. 6C), their structural interpretation is reported in the top-right side of Fig. S8. They were likely generated by the detachment of the anion of methoxy-carbonylated phosphoethanolamine, followed by the methanol neutral loss typical of mc-PE.

As described so far, several fragmentation pathways suggested that a methoxy-carbonyl moiety was linked to the NH_2_ group of PE 18:0/18:1. The same behaviour was observed for all the other major PE detected in the mitochondrial lipid extract of MEF cells. The characteristic neutral loss of methanol was systematically observed also when the [M−H]^−^ ions related to putative methoxy-carbonylated PE-O were examined, both under CID and HCD conditions.

## Discussion

As mentioned before, Garrett et al*.* related the occurrence of PE ethyl carbamates in the lipid extract of *E. coli* to the presence of the oxidative by-product phosgene (COCl_2_) in the chloroform used for lipid extraction^7^. Actually, the Authors observed that the deliberate addition of phosgene during the simulated extraction of a standard PE with chloroform and methanol led to generate the corresponding methylcarbamate. However, it was not clear why ethyl carbamates of PE were identified in the *E. coli* lipid extracts, even though methanol, not ethanol, was used as a co-solvent for lipid extraction^7^. Recently, the occurrence of a side reaction between the secondary amine group of ephedrine and phosgene resulting from photo-oxidated chloroform was proposed by Tsujikawa et al*.*^15^. Using gas chromatography-mass spectrometry (GC–MS), the Authors detected phosgene in ethanol-stabilized chloroform, but not in amylene (2-methyl-2-butene)-stabilized one (i.e., the type of chloroform used in our case for lipid extraction).

To assess the eventual presence of phosgene traces resulting from photo-oxidation reactions, the HPLC-grade chloroform used for lipid extraction during the present study was analyzed by gas chromatography coupled to electron ionization-mass spectrometry (see experimental details in the Methods section). The solvent was analyzed after being withdrawn from a just-opened bottle and after being kept for 10 days in a clear glass vial including an air-filled headspace and exposed to laboratory light, to enhance photooxidation. In particular, four diagnostic ions, detected at nominal *m/z* ratios 63, 65, 98 and 100, were considered for phosgene detection using a Multiple-Selective Ion Monitoring (Multi-SIM) acquisition. Actually, only in the case of purposely photoxidized chloroform a weak chromatographic peak could be recognized in the multi-SIM chromatogram, with relative intensities observed for the four ions in the SIM spectra being compatible with those expected for phosgene. The identification was further confirmed by the inspection of the Full-MS spectrum acquired in the elution interval identified in the multi-SIM trace. On the other hand, no evidence of the presence of phosgene could be obtained for chloroform withdrawn from a just-opened bottle.

As a further control, a mixture of standard PE 14:0/14:0 and PS 16:0/16:0 was extracted according to the Bligh and Dyer protocol using purposely photoxidized chloroform and perdeuterated methanol (CD_3_OD). Under these experimental conditions the methoxycarbonylation reaction was expected to lead to mc-PE 14:0/14:0 and mc-PS 16:0/16:0 including three D atoms. The extraction mixture was analysed by flow injection-ESI(−)-FTMS and, as described in detail in Section S1 and Fig. S9 of the Supplementary Information, the peak signals of [M−H]^−^ ions of the triply deuterated forms of mc-PE 14:0/14:0, at *m/z* 695.470, and mc-PS 16:0/16:0, at *m/z* 795.522, were almost negligible in the mass spectrum, compared to those of the corresponding precursors. This outcome showed that even using photo-oxidated chloroform (which was, obviously, not the one adopted for lipid extraction from MEF mitochondria), the phosgene-mediated generation of methyl carbamates of PE and PS appeared irrelevant. As a further confirmation of this scenario, we retrospectively reconsidered the HILIC-ESI-FTMS analyses performed in recent years in our laboratory on the chloroform/methanol lipid extracts obtained from other biological or food matrices, like yeast, HEPG cells, skeletal muscle mitochondria and microgreens of *Brassica* vegetables. As a result, very weak responses of mc-PE and/or mc-PS were always detected in those samples, definitely much lower than those obtained for mc-PE and mc-PS in the mitochondrial lipid extract of MEF.

A different route for the mc-PS and mc-PE generation in the latter was thus hypothesized, namely, an enzymatic reaction related to a bacterial contamination, based on the already discussed unexpected observation of ethyl carbamates of PE in *E. coli* extracts obtained using chloroform and methanol for lipid extraction^7^. Accordingly, the occurrence of an accidental bacterial contamination during the preparation of MEF mitochondrial samples was investigated. The first clue came from the unexpected identification of the bacterium *Campilobacter jejunii* during an RNA-sequencing experiment performed on mitochondrial pellets obtained from human fibroblasts grown with an aliquot of the same culture medium used for MEF cells. Retrospective PCR analysis was then performed on trypsin and PBS reagents and on media and cells that were investigated in the same period as MEF involved in the present study and resulted in a PCR-positive band for the *C. jejunii* bacterial ribosomal RNA 16S in the case of those media and cells; on the other hand, no band was observed on MEF cells cultured in 2019, as well as on trypsin and PBS reagents (see Fig. S10 of Supplementary Information). Although its origin was not clear, a bacterial contamination was thus ascertained for MEF whose mitochondrial lipid extracts had exhibited relevant amounts of mc-PE and mc-PS.

Interestingly, the lipid profile of *C. jejunii* was recently studied by Cao et al*.*^16^ and a PL class not recognized by the Authors, labelled as PX, was reported, in addition to typical PL classes, including PE and PS. A careful evaluation of their results evidenced a mistake in the interpretation made by the Authors of MS/MS data related to PX, i.e., the assignment of a CO_2_ loss (that would correspond to a − 44 shift in the nominal *m/z* ratio) instead of an ethanol loss for a peak signal nominally shifted by − 46 m*/z* units compared to the precursor ion. Consequently, PX were likely ethyl carbamates of PE, and, as in the case of *E. coli*^7^, no ethanol was ever used for the lipid extraction of *C. jejunii*. Although not explicitly, this finding provided additional evidence that carbamates of PE and PS can be formed in biological samples involving bacteria. Notably, the existence of a bacterial metabolic pathway leading to carbamates of compounds including amine groups is also supported by the generation of the methyl carbamate of a glycine molecule reported as a part of the complex biosynthesis of the natural antibiotic corallopyronin A by the *Corallococcus coralloides* myxobacterium^17^.

Based on these observations, we hypothesized that a contamination by *C. jejunii* might have been responsible for the unusual amounts of mc-PE and mc-PS found in the mitochondrial lipid extract of MEF. In order to find a further proof for this hypothesis, two new cultures of MEF cells were prepared using a carefully purified culture medium, prepared using gamma-irradiated fetal bovine serum filtered using disposable filter units with a pore size of 0.1 μm. When the corresponding mitochondrial lipid extracts were analyzed by HILIC-ESI(−)-FTMS, mc-PE and mc-PS were found to be barely detectable in both cases, just as it was in all samples previously analyzed in our laboratory. Indeed, the corresponding chromatographic bands could be recognized only after extracting ion currents referred to the *m/z* ratios of monoisotopic ions for major mc-PE and mc-PS, as inferred from the FTMS spectra of the PE and PS classes.

It is worth noting that the partial conversion of PE and PS in the corresponding methyl carbamates may significantly alter their quantitative data and even lead to problems in the identification of those species, unless a PL class-specific separation technique like HILIC is applied. For example, mc-PE and mc-PS might be isomeric with oxidized PE/PS including two additional O atoms and a further CH=CH moiety on their acyl chains. The approach proposed in this paper might thus be useful to preliminarily check if methyl (or, eventually, ethyl) carbamates of PE and PS are present in lipid extracts and then take proper countermeasures to minimize their formation. This control would be particularly important if the generation of those unusual PL was related to an unexpected bacterial contamination.

From a more general point of view, the existence of a metabolic pathway leading to the generation of methyl carbamates of PS and PE (and, more generally, of molecules including a primary amine group) in bacteria, apparently not investigated in detail so far in the literature, might deserve a more extended study, aiming at understanding its biological role.

## Methods

### Chemicals

LC–MS grade water, methanol, and acetonitrile, used for HILIC mobile phase preparation, HPLC-grade methanol, and chloroform, employed for lipid extraction, perdeuterated methanol (CD_3_OD), and reagent-grade ammonium acetate, used as mobile phase additive, were all purchased from Sigma-Aldrich (Milan, Italy). Standards of 1,2-dimyristoyl-sn-glycero-3-phosphoethanolamine (PE 14:0/14:0) and 1,2-dipalmitoyl-sn-glycero-3-phospho-l-serine (PS 16:0/16:0) were purchased from Avanti Polar Lipids (Alabaster, AL, USA).

### Culture of mouse embryonic fibroblasts and mitochondria isolation

Mitochondria subjected to lipid extraction during the present study were isolated from wild-type MEF cells kindly provided by Prof. Chan (California Institute of Technology, Los Angeles, USA). Cells were cultured at 37 °C in a humidified atmosphere with 5% CO_2_ in high glucose Dulbecco's Modified Eagle's Medium (Euroclone, Milan, Italy) supplemented with 10% fetal bovine serum (Euroclone, Milan, Italy), 50 U of penicillin G/mL, 50 μg/mL of streptomycin sulfate and 2 mM of l-glutamine (Sigma-Aldrich, Milan, Italy). Cell counting was performed using the Scepter™ Automated Cell Counter (Sigma-Aldrich, Milan, Italy), according to the manufacturer's instructions, in parallel to trypan blue visualization, adopted to exclude significant differences in live/dead trypsinised cells ratio among the tested cells.

Mitochondria from MEF cellular lines were isolated using Dounce homogenization, as described in the protocol of the mitochondria isolation kit for cultured cells (product 89874, purchased from Thermo Fisher Scientific, Rodano, Italy), with a modification aimed at obtaining a more purified fraction of mitochondria, with more than 50% reduction of lysosomal and peroxisomal contaminants. Specifically, after incubating cells (20 × 10^6^) with 800 mL of the kit Reagent A for 2 min on ice, Dounce homogenization was performed, then 800 mL of Reagent C were added, and the suspension was centrifugated for 10 min at 700*g* and 4 °C. The resulting supernatant was centrifugated for 15 min at 3000*g* and 4 °C, then the mitochondrial pellet was retrieved and washed with 500 mL of Reagent C. Finally, centrifugation at 12,000*g* at 4 °C was performed for 5 min and the resulting pellet was collected. Protein concentration related to mitochondria was determined using the BCA protein assay reagent (product 23227, Thermo Fisher Scientific, Rodano, Italy).

### Western blotting

Immunodetection of citrate synthase and β-actin was carried out in whole cells and in the mitochondrial fraction derived from MEF cells line. The cells were lysed in RIPA buffer, and twenty micrograms of total proteins relative to whole cells, previously lysed in RIPA buffer (product 89900, Thermo Fisher Scientific, Rodano, Italy) and mitochondria were treated with 10 mM Tris/HCl pH 6.8, 2% SDS, 5% β-mercaptoethanol, subjected to 15% SDS–polyacrylamide gel and subsequently transferred on nitrocellulose membrane. Western blot analysis was performed using antibodies against citrate synthase (product MA5-17264, Invitrogen, Waltham, MA, USA) and β actin (product MA5-15739, Invitrogen, Waltham, MA, USA). Antigen–antibody complexes were detected using anti-mouse IgG-coupled horseradish peroxidase (product 31430, Thermo Fisher Scientific, Waltham, MA, USA). Densitometric analyses of the relative bands was accomplished by using the Image Lab™ Touch software (Bio-Rad Laboratories, Hercules, CA USA).

### Enzymatic assay

The enzymatic activity of citrate synthase was measured by spectrophotometric assay following the absorption at 412 nm by the product thionitrobenzoic acid, which, in the presence of saturating concentrations of substrates acetyl-CoA, oxaloacetate, and 5,5′-dithiobis(2-nitrobenzoic acid), is a function of the activity of citrate synthase^18^. So doing, C.S. activity was measured in the cellular lysate and mitochondria using 30 μg of proteins for both.

### Detection of bacterial contamination in the culture medium of MEF

Since bacterial contamination was suspected in the culture medium adopted for the growth of MEF subsequently employed to obtain some mitochondria preparations (vide infra), a specific PCR test was performed to confirm the identity of the contaminating microorganism, which was presumed to be *Campilobacter jejunii*, according to preliminary RNA-based analyses. At this aim, 10 mL of sterile medium, of medium collected from cell cultures or of cell suspension were collected and centrifuged at 21,000*g* for 5 min. DNA was extracted from the pellets using the NucleoSpin Tissue kit (Macherey–Nagel, Dueren, Germany), following the manufacturer's support protocol for bacteria, which includes a pre-lyse step, in which samples are incubated with Proteinase K at 56 °C for 1 h. Primers for bacterial 16S were used to amplify the fragment of around 900 bp. The PCR conditions were: one cycle at 98 °C for 10 s; 35 cycles at 98 °C for 10 s, 55 °C for 5 s and 72 °C for 10 s; a final extension cycle at 72 °C for 10 s. The PCR was performed using Takara PrimeStar Max (Takara Bio Europe, Saint-Germain-en-Laye, France) and PCR products were separated by a 2% agarose gel. Primers were 16S Fw: GACTACCNGGGTATCTAATCC and 16S Rv: AGAGTTTGATCCTGGCTAAG.

### Lipid extraction from MEF mitochondria

Lipid extraction from MEF mitochondria was performed using the Bligh and Dyer protocol^19^, properly adapted to the specific samples. In particular, mitochondrial pellets were re-suspended into 800 µL of LC–MS water at room temperature, then 3 mL of a methanol/chloroform 2:1 (v/v) mixture were added; the resulting suspension was vortexed for 1 min and then left quiescent in the dark and in an ice bath for 1 h. At the end of this step, the suspension was centrifuged at 4500*g* for 10 min and a volume of 1 mL was subsequently withdrawn from the supernatant. Equal volumes (1.25 mL) of water and chloroform were added and, after vortexing for 1 min, the resulting mixture was centrifuged at 4500*g* for 10 min, to facilitate phase separation. The underlying, chloroform-rich, phase was subsequently collected and evaporated to dryness under a gentle nitrogen flux. Afterwards, the solid residue was re-dissolved in 100 µL of a methanol/chloroform 2:1 (v/v) mixture and the resulting solution was transferred into a screw-cap vial, whose headspace was saturated with nitrogen (to minimize lipid oxidation) before storage at − 20 °C until HILIC-ESI–MS analysis was performed.

### HILIC-ESI–MS instrumentation and operating conditions

HILIC-ESI–MS analyses of lipid extracts obtained from MEF mitochondria were performed using two LC–MS platforms, both including an Ultimate 3000 HPLC quaternary chromatographic system and, respectively, a *Q-Exactive* high-resolution Fourier-transform quadrupole-Orbitrap mass spectrometer (Thermo Fisher, West Palm Beach, CA, USA) and a *Velos Pro* low-resolution double stage linear ion trap mass spectrometer (Thermo Fisher, West Palm Beach, CA, USA). In both cases, a heated electrospray ionization source (Thermo Fisher, West Palm Beach, CA, USA) was used as the interface between liquid chromatography and mass spectrometry.

Chromatographic separations were performed using an Ascentis Express HILIC column (15 cm length, 2.1 mm internal diameter) packed with *core–shell* 2.7 µm silica particles (Supelco, Bellefonte, PA, USA) and preceded by a guard column (2 cm length, 2.1 mm internal diameter), packed with the same type of particles. A 0.3 mL/min flow rate, a 5 mL sample volume and a binary elution gradient based on an acetonitrile/water (97:3 v/v) mixture as phase A and a methanol/water (90:10 v/v) mixture as phase B, both containing ammonium acetate 2.5 mM, were adopted for lipid separation. The elution program was the following: 0–10 min) linear increase of B from 2 to 20%; 10–15 min) linear increase of B from 20 to 50%; 15–20 min) isocratic at 50% B; 20–25 min) return to 2% B; 25–30 min) column reconditioning at 2% B.

The parameters of the HESI interface and the ion optics of the *Q-Exactive* spectrometer, adopted for FTMS and FTMS/MS analyses, performed in negative polarity, were set as follows: sheath gas flow rate) 35 a.u.; auxiliary gas flow rate) 15 a.u.; spray voltage) − 2.5 kV; capillary temperature) 320 °C; S-lens RF level) 100. The spectrometer was operated at its maximum resolving power (140,000 at *m/z* 200) when FTMS spectra were acquired, using a 100–2000 m*/z* interval. During FTMS measurements the Orbitrap fill time was set to 200 ms and the Automatic Gain Control level was set to 3 × 10^6^. The spectrometer was calibrated daily by infusing, at a 5 mL/min flow rate, calibration solutions provided by the instrument manufacturer for negative polarity acquisitions. As a result, a mass accuracy always better than 5 ppm was achieved. FTMS/MS analyses were performed on selected precursor ions employing the Higher Energy Collisional Dissociation cell included in the *Q-Exactive* spectrometer. Specifically, the major isotopologue of each precursor ion was isolated in the spectrometer quadrupole and then fragmented into the HCD cell with a Normalized Collisional Energy equal to 28%. A lower resolving power (17,500 at *m/z* 200) was adopted for FTMS/MS acquisitions, to increase the acquisition rate.

The *Velos Pro* spectrometer was adopted for low-resolution MS/MS and MS^3^ analyses following HILIC separations. In this case, the parameters of the ion source and of the spectrometer ion optics were set as follows: sheath gas flow rate) 35 a.u.; auxiliary gas flow rate) 5 a.u.; spray voltage) − 2.5 kV; capillary temperature) 350 °C; S-lens RF level) 64.2. During MS experiments the linear ion trap fill time was set to 10 ms and the AGC level was set to 3 × 10^4^. MS^n^ spectra were acquired in negative ion mode using a 1 m*/z* unit wide window to isolate the monoisotopic peak of the precursor ion and setting the normalized collisional energy as 35%.

### Analysis of phosgene in chloroform by gas chromatography-mass spectrometry

A Clarus 680 gas chromatograph mounting an Elite-5MS chromatographic column (30 m × 0.25 mm i.d. × 0.25 mm) and coupled to a Clarus SQ8T quadrupole mass spectrometer (Perkin Elmer, Waltham, Massachusetts, USA) was adopted for GC–MS analyses of chloroform adopted for lipid extraction in the present study. The separations were performed under the following experimental conditions: injector temperature, 150 °C; injection method, split (1:20); injection volume, 1 mL; carrier gas, helium (flow 0.7 mL/min); temperature program: initial temperature 30 °C, increase up to 70 °C at a 6.7 °C/min increase, isothermal stage at 70 °C for 3 min. The transfer line between the gas chromatograph and the mass spectrometer was kept at a constant temperature of 250 °C. MS acquisitions were performed using electron ionization at 70 eV and 250 °C; spectra were acquired in the multiple single ion monitoring mode.

### Supplementary Information

Supplementary Information.

## Data Availability

Data can be shared upon request (contact: Prof. Ilario Losito, University of Bari “Aldo Moro”, Department of Chemistry, e-mail: ilario.losito@uniba.it).
