# Supplementary material for: Methyl carbamates of phosphatidylethanolamines and phosphatidylserines reveal bacterial contamination in mitochondrial lipid extracts of mouse embryonic fibroblasts
Source: Sci Rep. 2023 Aug 26;13:13972. doi: 10.1038/s41598-023-40357-5 (PMC10460386; doi:10.1038/s41598-023-40357-5)
Supplement: Supplementary file 1 — Supplementary Information. [file 41598_2023_40357_MOESM1_ESM.docx]

**Supplementary Information**

**Methyl carbamates of phosphatidylethanolamines and phosphatidylserines reveal bacterial contamination in mitochondrial lipid extracts of mouse embryonic fibroblasts**

Andrea Castellaneta^1^, Vito Porcelli^2^, Ilario Losito^1,3,*^, Serena Barile^2^, Alessandra Maresca^4^, Valentina Del Dotto^5^, Ludovica Sofia Guadalupi^1^, Cosima Damiana Calvano^1,3^, Valerio Carelli^4,5^, Luigi Palmieri^2,6^, Tommaso R.I. Cataldi^1,3^

*^1^Dipartimento di Chimica, ^2^Dipartimento di Bioscienze, Biotecnologie e Ambiente and ^3^Centro Interdipartimentale SMART - Università degli Studi di Bari Aldo Moro, via Orabona 4, 70126 Bari, Italy; ^4^IRCCS Istituto delle Scienze Neurologiche di Bologna, Programma di Neurogenetica, via Altura 3, 40139 Bologna, Italy, Italy; ^5^Dipartimento di Scienze Biomediche e Neuromotorie, Università degli Studi di Bologna, via Altura 3, 40139 Bologna, Italy; ^6^CNR-Istituto di Biomembrane, Bioenergetica e Biotecnologie Molecolari, Via Giovanni Amendola, 122/O, 70126 Bari, Italy*

**
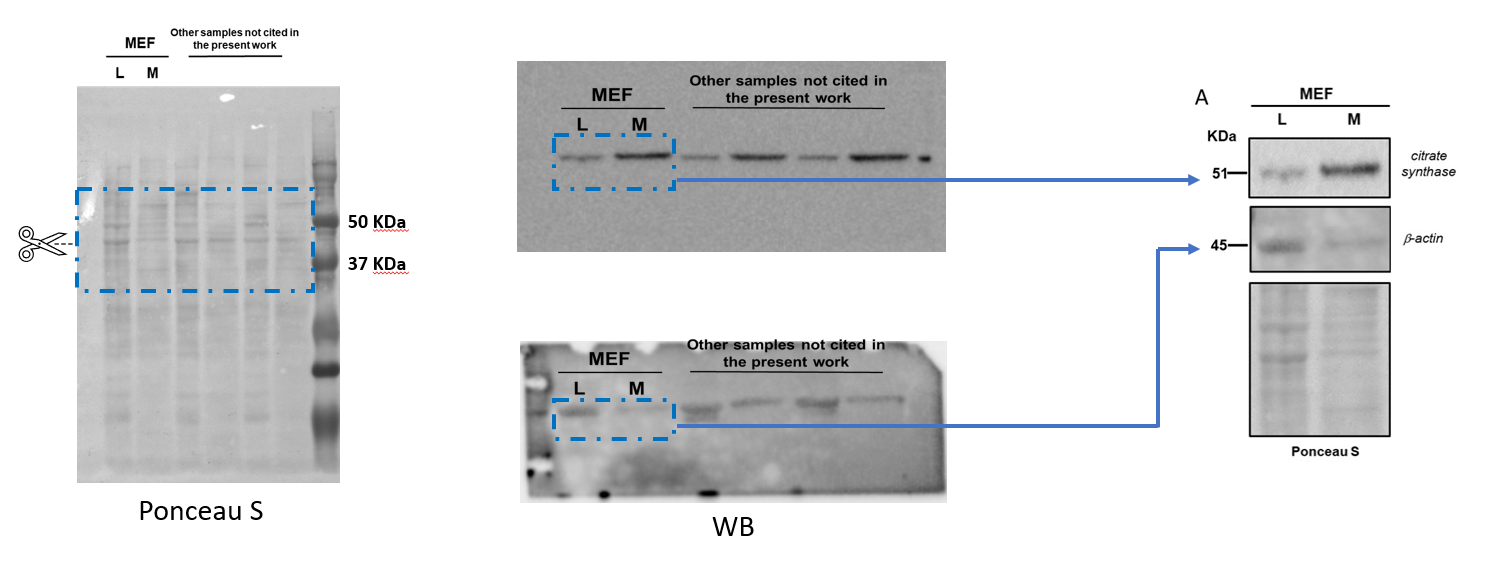
**

**Figure S1**. Details on the procedure adopted to obtain cropped gel images reported in panel A of Figure 1. The original gel, loaded with the cellular lysate (L) and the mitochondrial fraction (M) derived from a MEF cells line, along with other samples not involved in the present study, was transfered onto nitrocellulose membrane and stained with PonceauS. Afterwards, it was cut where indicated and the membrane was incubated with antibodies against citrate synthase and β-actin separately, as described in the main manuscript. Detailed views of the L and M lanes in the membrane after incubation with the two antibodies were reported in Figure 1A, along with the view of the same lanes in the section of the PonceauS-stained membrane obtained after cutting.


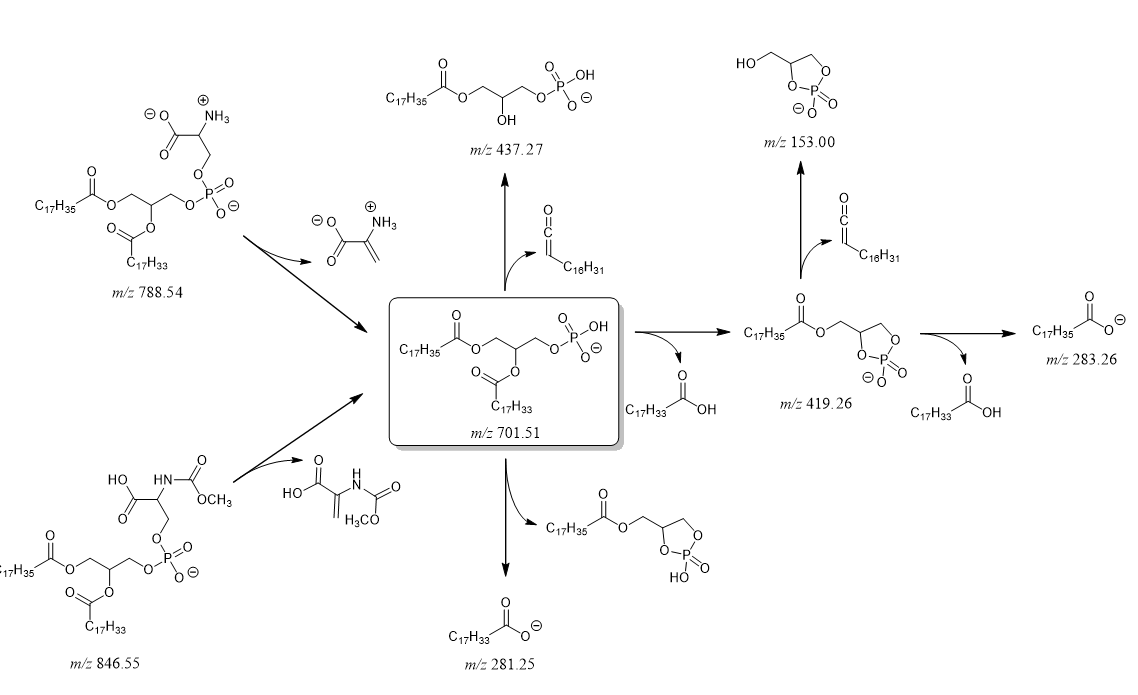


**Figure S2**. Main fragmentation pathways hypothesised to account for peak signals detected in HCD-MS/MS spectra of the [M-H]^−^ ions of PS 18:0/18:1 (*m/z* 788.54) and mc-PS 18:0/18:1 (*m/z* 846.55). Exact values, rounded off to the second decimal digit (to facilitate the comparison with experimental data shown in Figure 3) are reported for *m/z* ratios.


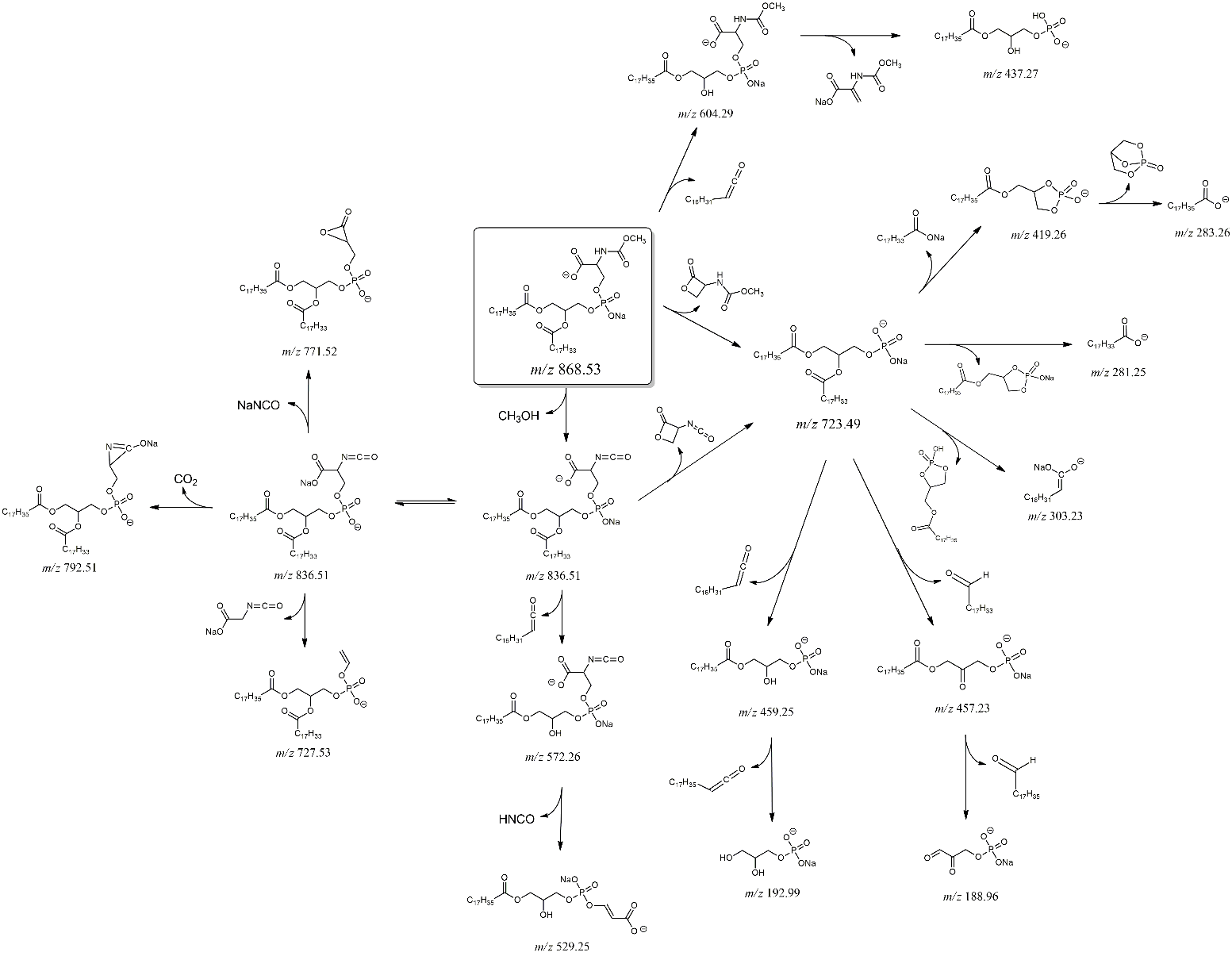


**Figure S3.** Fragmentation pathways hypothesized to explain the product ions detected in the MS/MS and MS^3^ spectra obtained for the [M-2H+Na]^−^ ion of mc-PS 18:0/18:1. Note that side chain fragmentations are shown only for the acyl chain linked to the sn-2 position of glycerol.


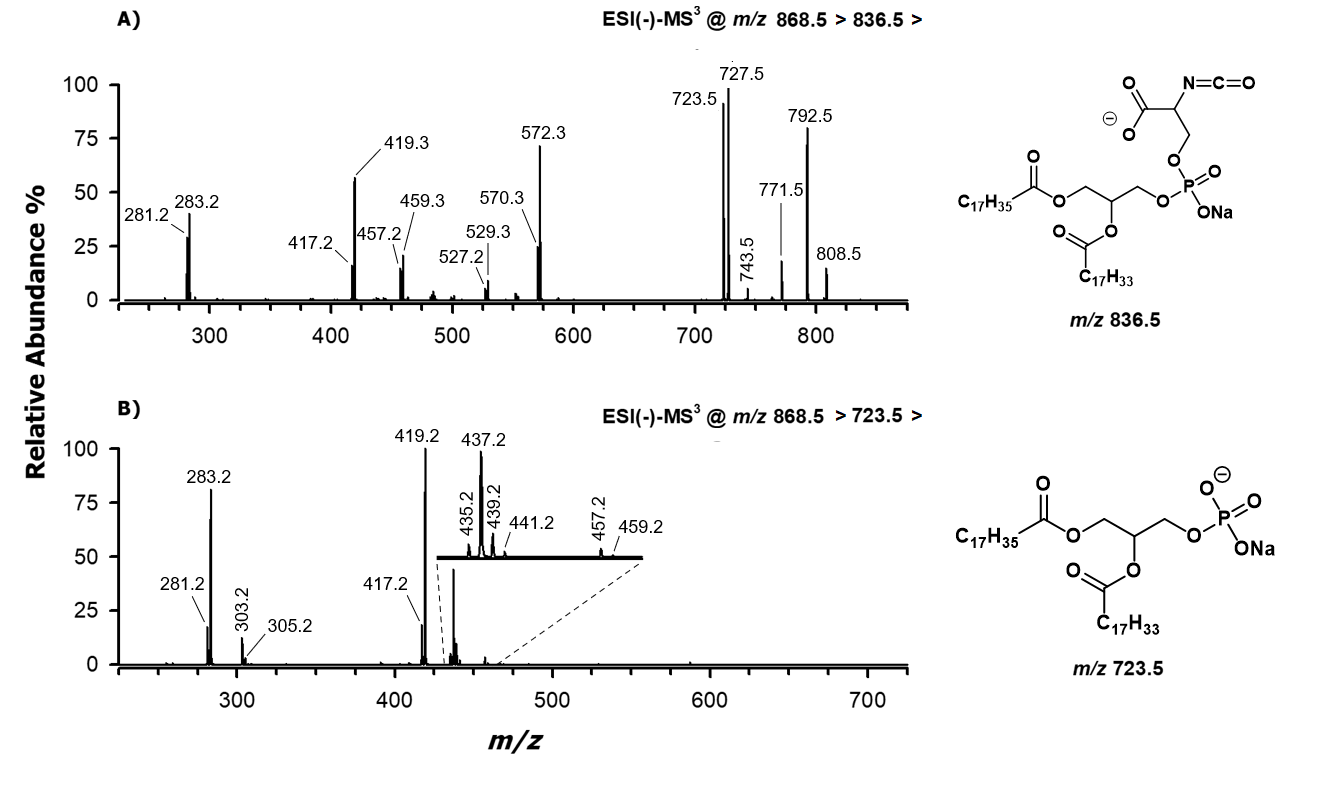


**Figure S4**. A) CID-ESI(-)-MS^3^ spectrum obtained for the product ion (*m/z* 836.5) generated by the neutral loss of methanol from the [M-2H+Na]^−^ ion (*m/z* 868.5) of mc-PS 18:0/18:1. B) CID-ESI(-)-MS^3^ spectrum obtained for the [M-2H+Na]^−^ ion of PA 18:0/18:1 (*m/z* 723.5), resulting from the corresponding ion of mc-PS 18:0/18:1 upon neutral loss of methoxycarbonyl-dehydro-serine. The hypothesised structures of both precursor ions are also shown. In the case of the *m/z* 836.5 ion an alternative structure, implying the interaction between the Na^+^ ion and the carboxylate moiety (with the negative charge located on the phosphate group) can be suggested.





**Figure S5**. Fragmentation pathways hypothesised to explain the detection of product ions with *m/z* 808.5 and 743.5 in the 868.5 > 836.5 > CID-MS^3^ spectrum obtained for the [M-2H+Na]^-^ ion of mc-PS 18:0/18:1 (see Figure S4A). Exact values, rounded off to the second decimal digit, are reported for *m/z* ratios.


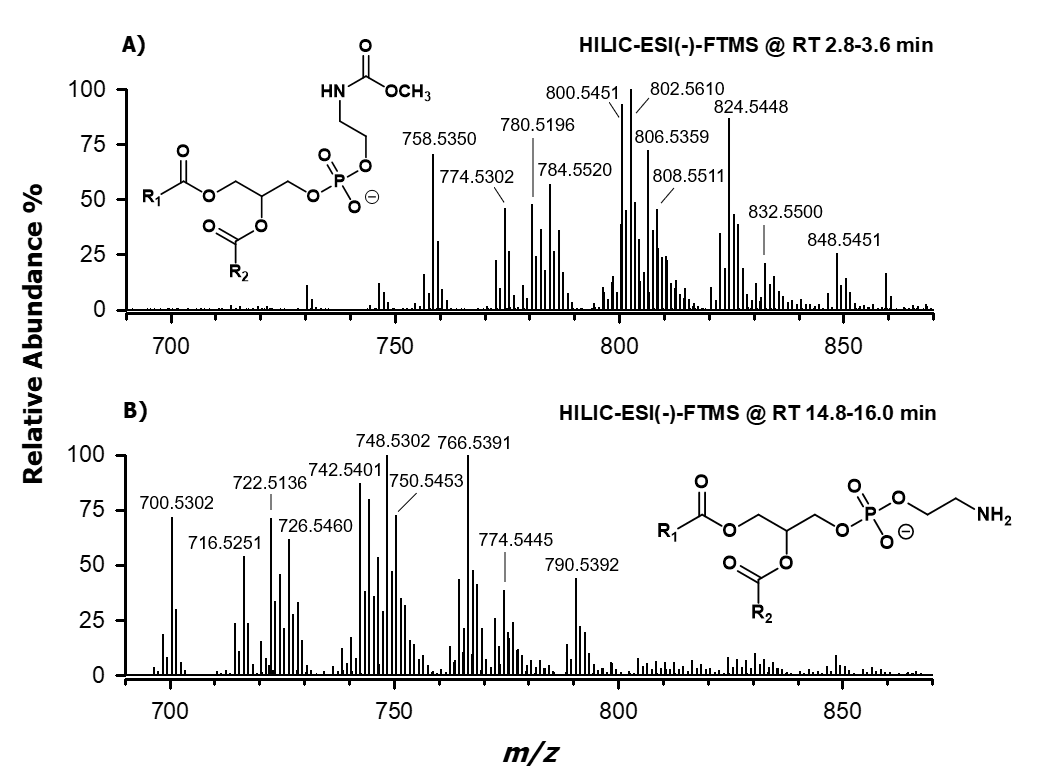


**Figure S6**. ESI(-)-FTMS spectra averaged under the bands assigned to mc-PE (A) and PE (B) detected in the TIC chromatogram shown in Figure 1. The labels refer to accurate *m/z* ratios measured for the most intense peak signals. The general structures for the [M-H]^-^ ions of mc-PE and PE are also shown.


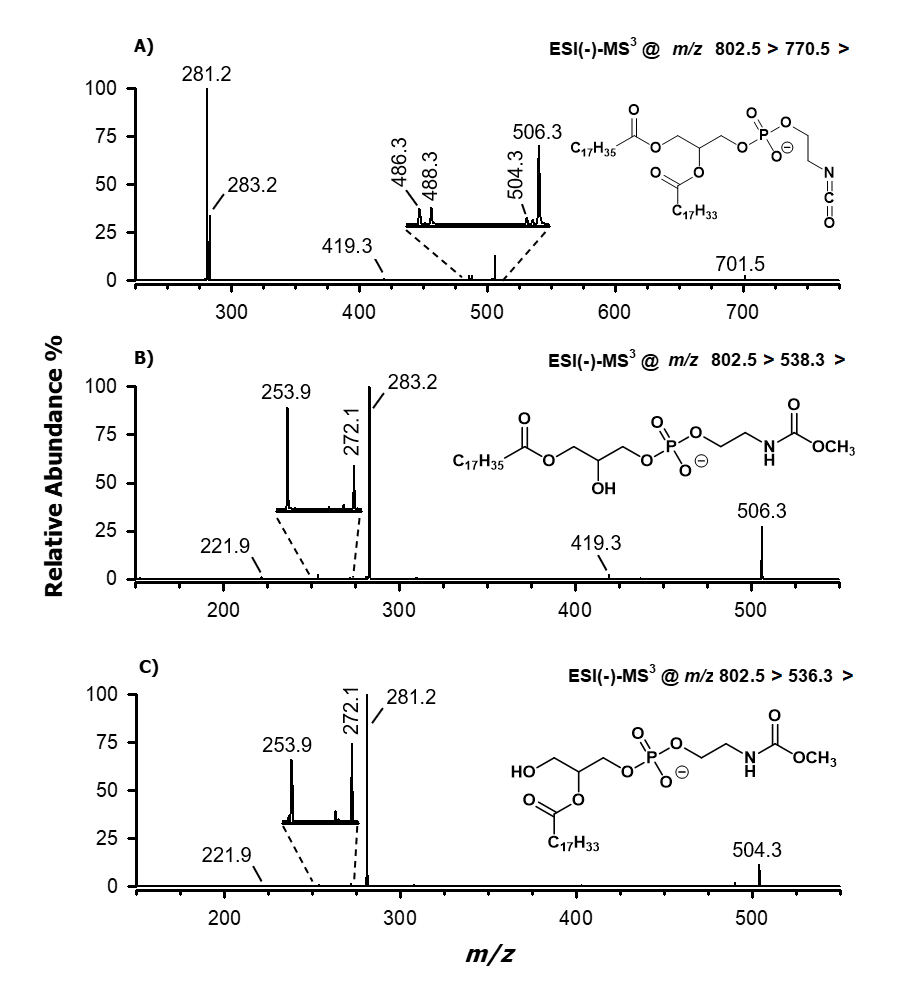


**Figure S7**. A) CID-ESI(-)-MS^3^ spectrum of the product ion (*m/z* 770.5, see the structure in the inset) generated by methanol neutral loss from the [M-H]^−^ ion of mc-PE 18:0/18:1 (m/z 802.5). B) CID-ESI(-)-MS^3^ spectrum of the [M-H]^−^ ion of mc-PE 18:0/0:0 (*m/z* 538.3), one of the *lyso* forms of mc-PE 18:0/18:1, generated upon 18:1 ketene loss from its [M-H]^−^ ion (see the structure in the inset). C) CID-ESI(-)-MS^3^ spectrum of the [M-H]^−^ ion of mc-PE 0:0/18:1 (*m/z* 536.3), the other *lyso* form of mc-PE 18:0/18:1, generated upon 18:0 ketene loss from its [M-H]^−^ ion (see the structure in the inset).


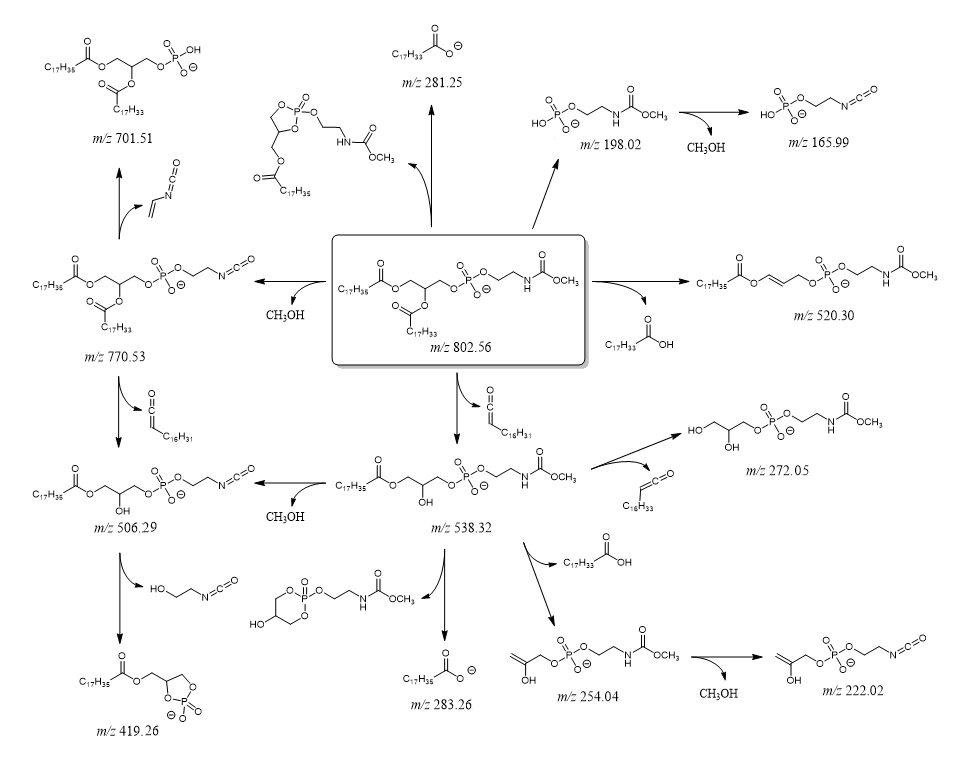


**Figure S8.** Fragmentation pathways hypothesized to explain the product ions detected in the MS^n^ (n = 2,3) spectra obtained for the [M-H]^−^ ion of mc-PE 18:0/18:1.

**Section S1. Extraction of standard PE and PS by the BD protocol involving photoxidized chloroform and perdeuterated methanol, followed by FIA-ESI(-)-FTMS and -FTMS/MS analysis.**

PE 28:0 (14:0/14:0) and the PS 32:0 (16:0/16:0) lipid standards were extracted by the same Bligh-Dyer protocol described in the Methods section but using perdeuterated methanol (CD_3_OD) and chloroform previously kept for 10 days in a vial including air in the headspace and exposed to laboratory light, to enhance the generation of phosgene. CD_3_OD was used to verify the generation of carbamates of standard PE and PS including a CD_3_ group instead of a CH_3_ one, thus exhibiting a +3 Da mass shift compared to the regular methyl carbamates.

1 mL of a 100 μg/mL chloroform solution of PE 28:0 and PS 32:0 was subjected to solvent evaporation under nitrogen flux. The dry residue was resuspended in 800 μL of water and 2 mL of a CD_3_OD/CHCl_3_ 2:1 v/v mixture, in which photo-oxidized chloroform was used. Thereafter, the phase separation was induced by the addition of 1.25 μL of photo-oxidized chloroform and 1.25 μL of water. The chloroform-rich phase was withdrawn and subjected to solvent evaporation. The dry residue was dissolved in 1 mL of a CH_3_OH/CHCl_3_ 2:1 v/v solution. Regular chloroform was used at this stage and non-deuterated methanol was adopted to ensure the replacement of all the possible exchangeable deuterium atoms with hydrogen atoms in the structure of the extracted lipid species. The solution was diluted by a 1:2 factor and subjected to flow-injection analysis (FIA) by injecting the sample at a 50 μL/min flow in a 150 μL/min flow of a mixture including 25% of phase B and 75% of phase A (v/v) used for HILIC-ESI-MS analyses (see the Methods section), directed to the ESI source. The FIA-ESI(-)-FTMS spectrum of the sample solution is shown in **Figure S9A**. Here, the signals of the PE 28:0 and PS 32:0 [M-H]^−^ ions are recognizable at *m/z* 634.446 and *m/z* 734.499. On the other hand, the signal at *m/z* 647.466, corresponding to the [M-H]^−^ ion of a PA 32:0, can be attributed to the partial in-source fragmentation of PS 32:0. As evidenced in the magnified regions of the mass spectrum, all the signals referring to the PE and PS methyl carbamates showed very low intensities. In particular, both the [M-H]^−^ ions of regular and deuterated mc-PE (*m/z* 692.451 and *m/z* 695.470) and mc-PS (*m/z* 792.456 and *m/z* 795.522) were identified as very weak peak signals in the MS spectrum. Notably, the formation of regular methyl carbamates could happen only when the lipid residue was redissolved in the CH_3_OH/CHCl_3_ 2:1 v/v mixture. This indicated basal phosgene contamination of the chloroform that is regularly employed for lipid extraction in our laboratory, which led to a further reaction of PE and PS to form methyl carbamates when non-deuterated methanol was introduced. As apparent from **Figure S9A**, the existence of methyl carbamates was in any case very limited, almost negligible, if compared to that observed in the mitochondrial lipid extracts of MEF.

The fragmentation of deuterated mc-PE 28:0 in the HCD cell of the quadrupole-Orbitrap spectrometer led to the FTMS/MS spectrum shown in **Figure S9B**. Once the signals related to the isobaric interference due to the M+3 isotopologue of the [M-H+Na+Cl]^−^ ion adduct of PE 28:0 were recognized, the MS/MS spectrum exhibited fragmentations typical of mc-PE. In particular, the loss of deuterated methanol (nominal molecular mass 35 Da) was recognizable both from the precursor ion (*m/z* 660.424←695.469) and from its product ions resulting from ketene and fatty acid neutral losses of acyl chains (*m/z* 450.226← 485.271and *m/z* 432.215←467.260). Additionally, the *m/z* ratio of the polar head-related product ion containing the methanol residue in the carbamate molecule showed a nominal increase of 3 Da (see the peak signal at *m/z* 201.063 in **Figure S9B**). Conversely, the polar head-related ion emerging from the loss of such residue maintained the expected *m/z* ratio (*m/z* 165.991).

As expected, the FTMS/MS spectrum of the [M-H]^−^ ion of triply deuterated mc-PS 32:0 (*m/z* 795.5). shown in **Figure S9C**, mirrored the one of the corresponding phosphatidic acid (PA 32:0), since the neutral loss of the dehydrated serine methyl carbamate, including the three D atoms, was the main fragmentation event involving the polar head. No [M-2H+Na]^−^ adduct could be isolated and fragmented for both the regular and deuterated mc-PS 32:0 in this case.


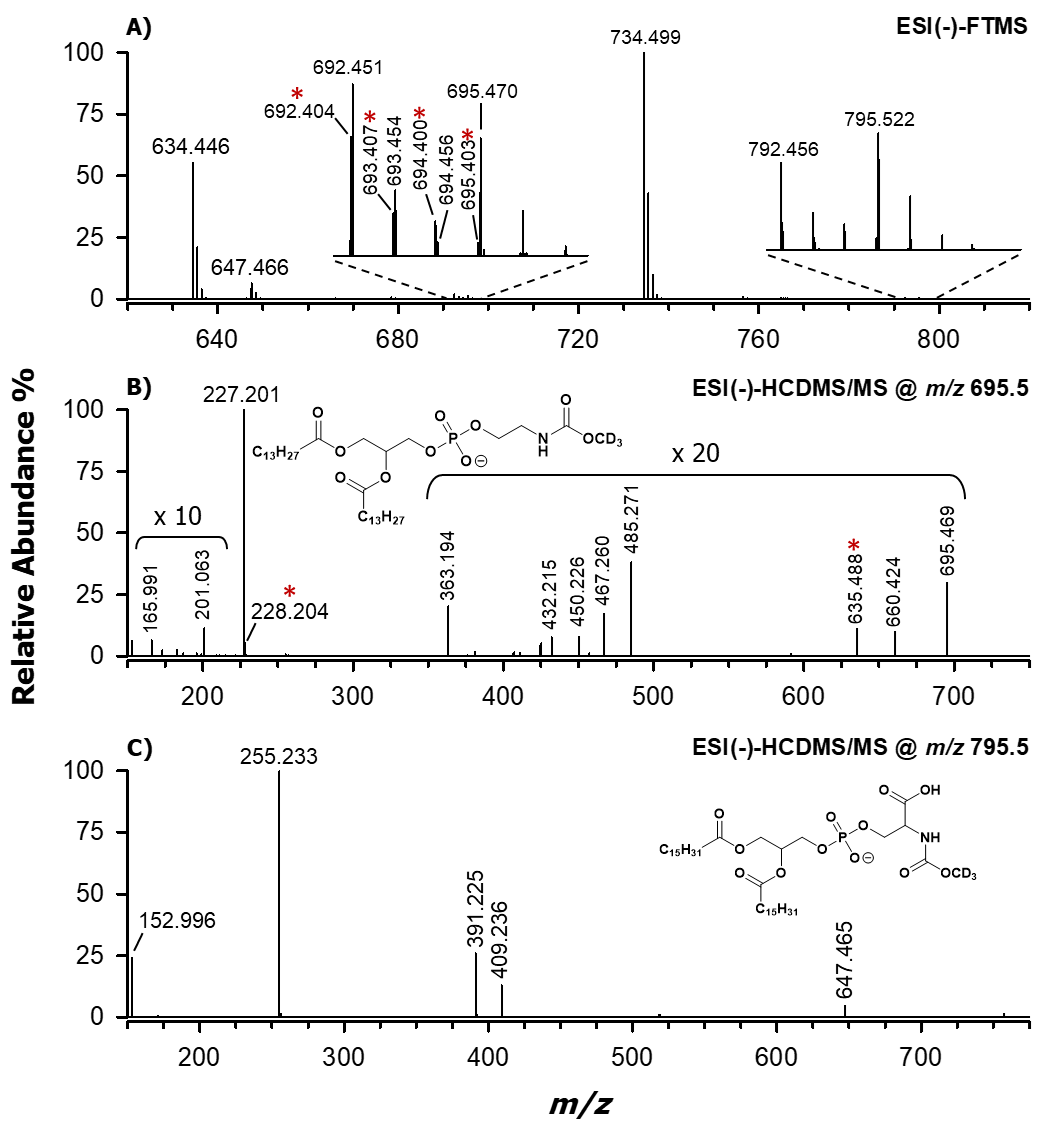


**Figure S9.** A) FIA-ESI(-)-FTMS spectrum of a PS 32:0 (16:0/16:0) and PE 28:0 (14:0/14:0) standard mixture extracted with the Bligh-Dyer protocol using photo-oxidized chloroform and perdeuterated methanol (CD_3_OD), as described in **Section S1**. The magnified spectral regions show the low-intensity peak signals due to [M-H]^-^ ions of triply deuterated mc-PE (*m/z* 695.470) and mc-PS (*m/z* 795.522), along with those of corresponding non-deuterated forms (*m/z* 692.451 and *m/z* 792.456). In particular, the isotope pattern of the non-deuterated mc-PE was entangled with the one of the [M-H+Na+Cl]^−^ adduct of PE 28:0. The signals referring to the latter ion were labelled with red asterisks. The identity of this ion was further confirmed by the accordance of the observed isotope ratios with those expected from the presence of a chlorine atom (data not shown). Even though the two isotope patterns were fully resolved when the Orbitrap mass analyzer was operated at its maximum resolving power (*i.e.*, 140000 at *m/z* 200), the interference of the M+3 isotopologue of the PE 28:0 [M-H+Na+Cl]^−^ ion (*m/z* 695.403) in the MS/MS spectrum of the deuterated mc-PE [M-H]^−^ ion (*m/z* 695.470) had to be considered, due to an inherent limitation in the isolation of precursor ions in the quadrupole analyzer of the *Q-Exactive* spectrometer.

B) ESI(-)-HCD-MS/MS spectrum of the triply deuterated mc-PE 28:0 [M-H]^−^ ion. All the signals labelled with the red asterisk refer to fragments related to the isobaric M+3 isotopologue of the PE 28:0 [M-H+Na+Cl]^−^ ion. C) ESI(-)-HCD-MS/MS spectrum of the triply deuterated mc-PS 32:0 [M-H]^−^ ion.


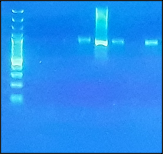


900 bp

500 bp

1000 bp

100 bp

1

2

3

4

5

6

7

8

**Figure S10**. PCR analysis showing an agarose electrophoresis separation of the PCR amplified fragment of 900 bp on the bacterial 16S of *C. jejunii*. Lanes are referred to the following samples: 1) reaction without DNA (blank sample); 2) trypsin reagent; 3) PBS reagent; 4) medium used for the growth of HeLa cells, in the same period of contaminated MEF; 5) medium used for the growth of MEF whose mitochondria lipid extracts included mc-PE and mc-PS; 6) medium used for the growth of human retinal pigment epithelial-1 (RPE1) cells, in the same period of contaminated MEF; 7) medium withdrawn from MEF frozen in 2019; 8) a sterile medium prepared with reagents used during the same period of contaminated MEF, HeLa and RPE1 cells cultures. The occurrence of the bacterial 16S is particularly relevant in the medium used to grow MEF whose mitochondria lipid extracts included mc-PE and mc-PS (lane 5).
